# Digestive tract morphology and enzyme activities of juvenile diploid and triploid Atlantic salmon (*Salmo salar*) fed fishmeal-based diets with or without fish protein hydrolysates

**DOI:** 10.1371/journal.pone.0245216

**Published:** 2021-01-11

**Authors:** Silvia Martínez-Llorens, Stefano Peruzzi, Inger-Britt Falk-Petersen, Sergio Godoy-Olmos, Lars Olav Ulleberg, Ana Tomás-Vidal, Velmurugu Puvanendran, Derrick Kwame Odei, Ørjan Hagen, Jorge M. O. Fernandes, Malcolm Jobling

**Affiliations:** 1 Aquaculture and Biodiversity Research Group, Institute of Science and Animal Technology (ICTA), Universitat Politècnica de València, València, Spain; 2 Faculty of Biosciences, Fisheries and Economics, UiT - the Arctic University of Norway, Tromsø, Norway; 3 Production Biology, Nofima AS, Tromsø, Norway; 4 Faculty of Biosciences and Aquaculture, Nord University, Bodø, Norway; Universidade de Vigo, SPAIN

## Abstract

Triploid, sterile Atlantic salmon (*Salmo salar*) could make a contribution to the development of the farming industry, but uncertainties about the performance and welfare of triploids have limited their adoption by farmers. In this study, we compared the ontogeny of digestive tract morphology and enzyme activities (pepsin, trypsin, chymotrypsin, alkaline phosphatase and aminopeptidase) of diploid and triploid Atlantic salmon. Fish were fed diets based on fishmeal (STD) or a mix of fishmeal and hydrolysed fish proteins (HFM) whilst being reared at low temperature from start-feeding to completion of the parr-smolt transformation. Fish weights for each ploidy and feed combination were used to calculate thermal growth coefficients (TGCs) that spanned this developmental period, and the data were used to examine possible relationships between enzyme activities and growth. At the end of the experiment, faeces were collected and analyzed to determine the apparent digestibility coefficients (ADCs) of the dietary amino acids (AAs). Digestive tract histo-morphology did not differ substantially between ploidies and generally reflected organ maturation and functionality. There were no consistent differences in proteolytic enzyme activities resulting from the inclusion of HFM in the diet, nor was there improved digestibility and AA bioavailability of the HFM feed in either diploid or triploid fish. The triploid salmon had lower ADCs than diploids for most essential and non-essential AAs in both diets (STD and HFM), but without there being any indication of lower intestinal protease activity in triploid fish. When trypsin-to-chymotrypsin activity and trypsin and alkaline phosphatase (ALP) ratios (T:C and T:ALP, respectively) were considered in combination with growth data (TGC) low T:C and T:ALP values coincided with times of reduced fish growth, and vice versa, suggesting that T:C and T:ALP may be used to predict recent growth history and possible growth potential.

## Introduction

Triploid Atlantic salmon (*Salmo salar*) may have an important role to play in the sustainable expansion of the aquaculture industry in Norway and other salmon-producing countries. The use of functionally sterile fish has the potential to improve somatic growth, survival and flesh quality while mitigating ecological impacts on wild stocks in the event of accidental farmed fish escapes [1]. Despite this, uncertainties regarding the performance of cultured triploid stocks have hindered the widespread adoption of triploid salmon by the industry. The salmon-farming industry in Tasmania (Australia) is an exception, where triploid salmon are farmed to circumvent early sexual maturation of fish as grilse [2].

A number of studies have focused on the physiological and morphological consequences of triploidy in farmed fish, including Atlantic salmon (see reviews by [3–5]). Diploid and triploid Atlantic salmon show differences in the gross morphology of the digestive system [6] and in culturable gut microbiota [7] with potential consequences for nutrient utilization, growth and health. A recent analysis of the gut microbiome of diploid and triploid salmon reported similarities in the microbial communities of the fish at early life stages [8]. Little is known about the effects of feed formulations on digestive tract structure and function of triploid Atlantic salmon, but several studies carried out on diploid salmon have shown dietary effects on gut mucosal structure (e.g [9–14]). Information about the nutritional requirements of triploids is incomplete, but it is known that supplementation of feeds with histidine and phosphorus may reduce the incidence and severity of eye cataracts and skeletal anomalies, respectively [15–18]. In particular, triploid salmon require more dietary phosphorus than diploids during early development to achieve comparable bone mineralization [19]. Also, there are indications that triploid salmon parr may have different micronutrient requirements and metabolic responses to dietary supplementation than their diploid counterparts when fed low levels of marine ingredients [20,21].

Studies on fish digestive enzymes have increased knowledge about nutrient utilization [20,22–24]. Variations in proteolytic activity, for example, may be related to feed conversion efficiency and growth via effects on nutrient digestion, transport and absorption [25]. Several factors can modulate the intestinal enzymatic profile, such as the source, quality and concentration of dietary nutrients [26], ploidy [27] and fish developmental stage [13]. Enzyme activity in the digestive tract can be considered as an indicator of digestive capacity and nutritional status [28]. Understanding changes in the digestive system of triploid salmon that occur during development may give important pointers for improving feed formulation.

Hydrolysed fish protein (HFM) contains free amino acids (AAs) and small peptides that can stimulate the secretion of proteolytic enzymes and modulate their activity [29–35], and free AAs and peptides may also be absorbed more rapidly than the products of enzymatic breakdown of intact dietary proteins [36,37]. Biofunctional properties of HFM and their active compounds in promoting digestive activity, feed intake and effciency, and fish immunity [38] might have a positive effect on the growth (and survival) of triploid fish.

The focus of the present investigation is to compare digestive tract morphology and enzyme activities of juvenile diploid and triploid Atlantic salmon. The fish were fed either a standard salmon feed or one with hydrolysed fish proteins (see review by [31]), thought to be suitable for triploid Atlantic salmon [39].

## Materials and methods

This study was carried out in accordance with the Norwegian regulations for use of animals in experiments and was approved by the Norwegian Committee on Ethics in Animal Experimentation via project licence (Permit ID 8180) issued by the Norwegian Food Safety Authority (Mattilsynet, FOTS). The growth trial was carried out in an approved facility (Tromsø Aquaculture Research Station, FOTS licence nr. 110) by trained and licensed personnel. Terminal measurements were performed on fish euthanized with an overdose of anaesthetic (Benzocaine, 120 mg L^-1^). All efforts were made to minimize experimental fish number and fish suffering.

### Experimental fish and setup

**Fig 1:**
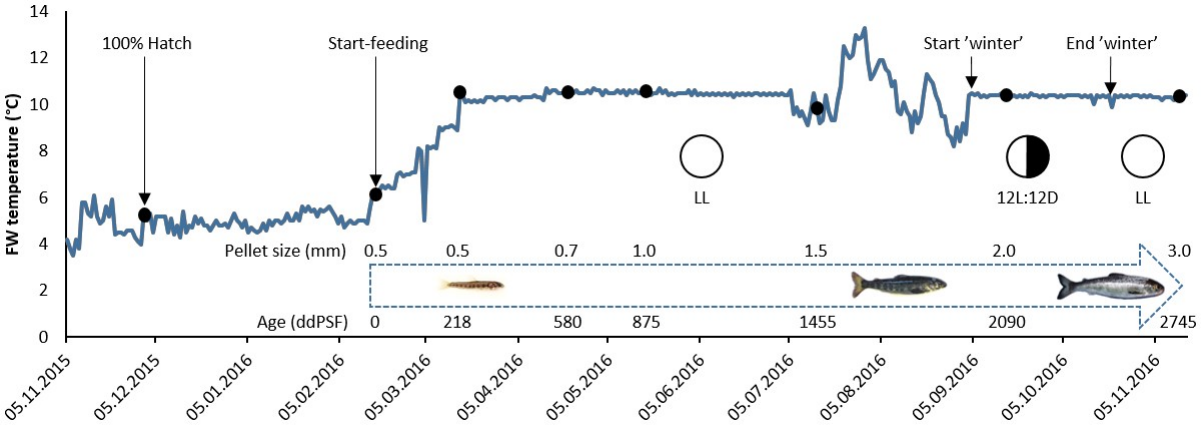
Overview of rearing conditions and main operations during the trial. Black dots indicate sampling points where 25 fish per tank were weighed and measured. Arrows indicate other events or operations. FW = freshwater; LL = constant light; 12L:12D = light regime used during ‘winter’ stimulation. Pellet size (mm) employed over the whole trial and fish age in degree-days post start-feeding (ddPSF) at each sampling point (0–2745 ddPSF) are shown. Figure adapted from [39].

This study was run in parallel with the one described in [39] where details about protocols for fertilization, triploidisation, and rearing are provided. Briefly, groups of diploid and triploid fish (n = 17 families/ploidy) were reared separately in triplicate tanks and fed diets based on fish meal (STD) or a mix of fishmeal and hydrolysed fish proteins (HFM) whilst being reared at low temperature from startfeeding to completion of the parr-smolt transformation. Feed (pellet size: 0.5–3.0 mm) was delivered via electrically driven disc feeders programmed to supply 6–9 meals each day, and the amount of feed provided was always in excess of that consumed. An overview of the rearing conditions and main events or operations carried out during the trial is provided in Fig 1.

At 580, 875, 1168, 1455, 1888, 2090, 2454 and 2745 degree-days post start-feeding (ddPSF), 25 fish were removed from each tank, euthanized and weighed (Fig 1). A detailed description of the sampling for histology and enzyme assays is given in Table 1. Treatment mean weights for each ploidy (2n, 3n) and feed (STD, HFM) combination were used to calculate thermal growth coefficients (TGC) for periods between sampling points. TGC was calculated using the formula given in Cho [40]:

$$TGC=1000\times \frac{\sqrt[{3}]{MW_{1}}-\;\sqrt[{3}]{MW_{0}}}{\sum degree-days}$$

where MW_0_ and MW_1_ are mean fish weights at the start and end of a growth period. TGC was chosen as the growth metric because it compensates for size and temperature effects on growth, thereby making growth comparisons easier than when using other growth metrics, such as specific growth rate (SGR).

**Table 1:** Sampling times given in weeks and degree-days post start-feeding (ddPSF).

Stage/ Weeks after start-feeding	ddPSF	Samples for digestive enzymes*	Samples for histology	Morphometric measurements*
**Hatching**	----	----	Whole fish	----
**Start-feeding**	0	----	Whole fish	----
**4 weeks after start-feeding**	218	----	Whole fish	----
**9 weeks after start-feeding**	580	----	Whole fish	----
**13 weeks after start-feeding**	875	ST and intestine	Whole fish	----
**21 weeks after start-feeding**	1455	ST and intestine	Intestine	AI, PI
**30 weeks after start-feeding**	2090	ST and intestine	Intestine	AI, PI
**38 weeks after start-feeding**	2745	ST, PC, AI, MI, PI	Intestine	AI, PI

*ST: Stomach, PC: pyloric caeca, AI: anterior intestine, MI: mid-intestine, PI: posterior intestine.

TGC data were used to look for possible relationships between enzyme activities and growth using simple linear regression for analysis, where appropriate. We sampled fish for the measurement of enzyme activities on four occasions (Table 1). This meant that we could combine the enzyme activity data with TGC recorded for the fish in the periods immediately prior to (e.g. 580–875 ddPSF) and after sampling (e.g. 875–1168 ddPSF), and for the period that spanned both prior and subsequent growth (e.g. 580–1168 ddPSF). Thus, we had data for four sampling points for enzyme analysis and ten growth periods covering diploid and triploid salmon fed either STD or HFM feed. We carried out the analyses using data for specific activities of trypsin and alkaline phosphatase (ALP), trypsin-to-chymotrypsin ratio (T:C), and ratio between trypsin and ALP activities.

### Histological preparations and examinations

**Fig 2:**
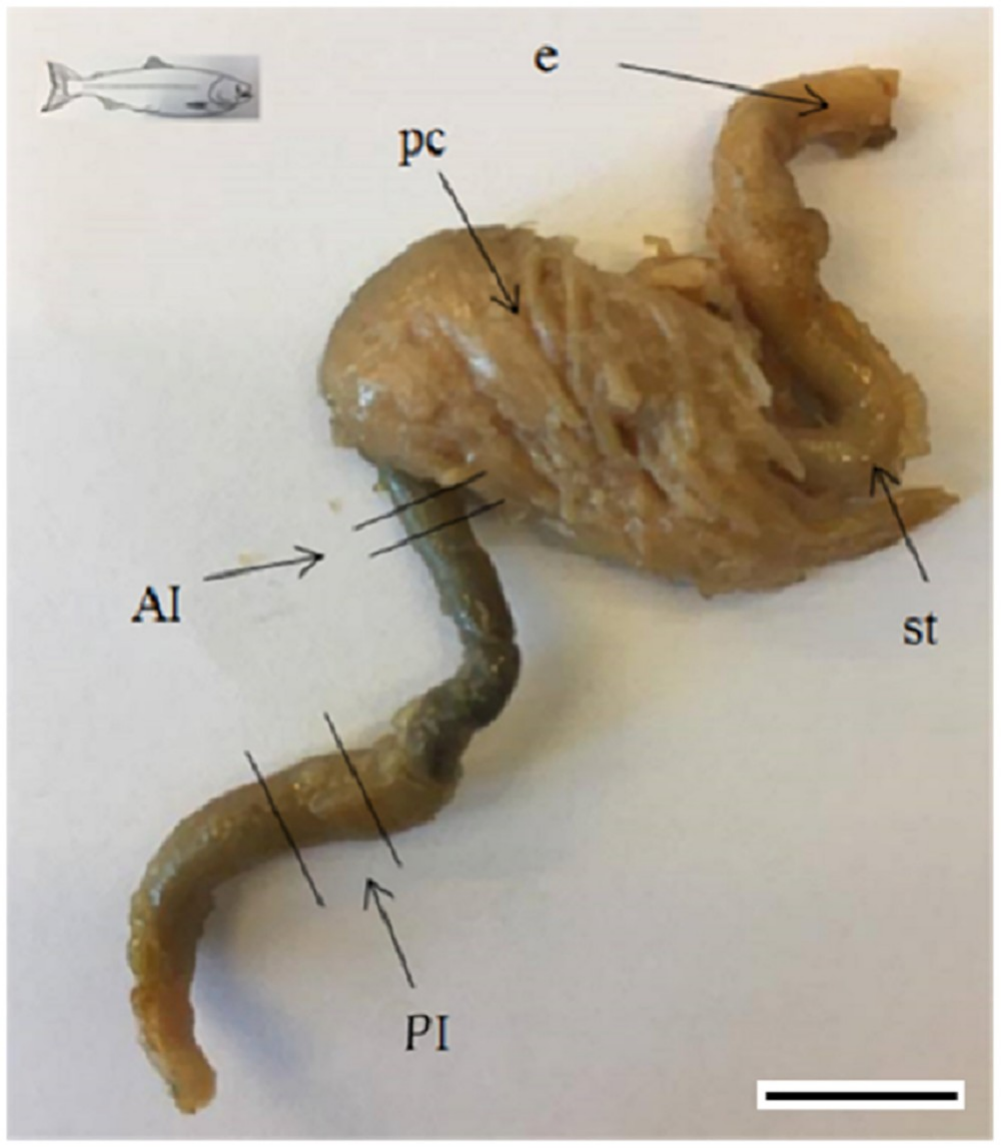
The complete gastrointestinal tract of Atlantic salmon divided into different parts. AI. anterior intestine; PI. posterior intestine; e. esophagus; pc. pyloric caeca; st. stomach. Scale bar represents 1 cm.

Fish sampled at different times were fixed in 10% buffered formalin (v/v) for at least 48 hours and transferred to 70% ethanol (v/v) for storage until being prepared for histological study (Table 1). At hatch and at start-feeding 20 diploid and 20 triploid salmon were sampled and fixed as whole fish. At 4 weeks after start-feeding (218 ddPSF) until 13 weeks after start-feeding (875 ddPSF) 5 fish from each tank were fixed as whole fish, while at 21 weeks after start-feeding (1455 ddPSF) until the end of the experiment (2745 ddPSF) gastrointestinal tracts from 5 individuals per tank were fixed. The fish were not fasted prior to sampling. The gastrointestinal tract of juvenile Atlantic salmon, including the parts taken for histological examination, is shown in Fig 2.

All samples were transferred to standard plastic cassettes, dehydrated, embedded in paraffin wax and sectioned at 5 μm on a rotary microtome (Leica RM 2255). The sections were stained (haematoxylin and eosin, H&E) and examined using a Leica DM 2000 LED light microscope (Leica Microsystems, Wetzlar, Germany) equipped with a Leica DFC 295 Digital Colour Camera. Photographs of sections were processed using the Leica software application suite (LAS).

Histological examination of the digestive tract was carried out on longitudinal sections of whole fish (the newly hatched and 0–13 weeks/0-875 ddPSF individuals) and cross sections of the anterior and posterior intestine from larger fish (Table 1). In larger juveniles, a segment cut immediately after the end of the pyloric region represented the anterior intestine (AI), and a similar segment cut at the fore part of the posterior intestine (darker colour and thicker diameter) represented the posterior intestine (PI) (see [41,42]), (Fig 2).

Histological comparisons of the whole digestive tracts of diploid and triploid salmon were performed for the earliest stages, from hatch/0 ddPSF to 580 ddPSF (samples from 875 ddPSF were discarded due to poor quality). Morphometric measurements of the intestinal mucosa and muscularis were compared at three sampling points (1455, 2090 and 2745 ddPSF) by measuring width and height of the intestinal folds, number of folds and thickness of the muscle layers under the microscope between 5x and 40x magnification. Measurements of the anterior and posterior intestinal fold heights (from tip of intestinal fold to basal cells) and widths of 10 different folds per individual section were done for the above mentioned sampling points (three individuals per tank, 9 individuals per diet group and ploidy) [39]. Ten measurements per section were performed for the thickness of the intestinal wall. Numbers of mucus cells in two complete folds per section were counted. The number of intestinal folds was determined from two cross sections of the intestine.

### Diets and digestibility measurements

The formulations and chemical compositions of the STD and HFM diets (Skretting AS, Stavanger, Norway) are provided in Tables 2 and 3.

**Table 2:** Formulation and composition of the STD and HFM diets (G.Reisen, Skretting AS).

	STD (Only fishmeal)			HFM (With CPSP)		
***Ingredients***						
**Pellet size (mm)**	0.5/0.7/1.0	1.2	1.5/2.0/3.0	0.5/0.7/1.0	1.2	1.5/2.0/3.0
**Wheat**	7.2	6.1	6.9	5.4	5.5	6.9
**Wheat gluten**	10	10	10	10	10	10
**SPC**	14.4	16.7	17.9	14	16.2	16.7
**NA Fishmeal**	55	55	50	30	30	27.5
CPSP*	0	0	0	25	25	22.5
**Fish oil Nordic**	11	10.8	11.6	9.4	9.2	10.2
**Water/Moisture**	0	0	0.4	1.6	0.9	1.5
Yttrium Premix**	0	0	0.1	0	0	0.1
**Premix (min, Vit, AA)**	2.4	1.4	3	4.7	3.3	4.6
***Total***	100	100	100	100	100	100
***Chemical composition (%)***						
**Dry matter**	92.1	92.5	92.8	92.1	92.5	92.9
**Crude protein**	55.9	56.8	56.0	60.3	59.0	56.9
**Crude fat**	17.7	18.3	19.1	17.3	18.8	19.6

SPC = Soy Protein Concentrate.

*CPSP = Fish meal hydrolysate Special-G^®^ (SoproPêche. Boulogne-sur-Mer. France).

**Marker Yttrium used in 3.0mm diets only. Buffer capacity (mEq/ g needed to reach pH 3.0.) was 1.3 and 0.9 in STD and HFM diets, the initial pH of these diets being 6.2 and 6.0, respectively.

**Table 3:** Amino acid profile of standard (STD) and experimental diet (HFM) expressed as g individual AA per 100 g diet dry matter. Data refer to 3mm pellet size diets used at the end of the experimentation (2745 ddPSF).

	Diet		
(g AA/100 g diet)	AA*	STD	HFM
***Essential Amino acids***			
**Arginine**	*ARG*	3.05	3.11
**Histidine**	*HIS*	1.39	1.39
**Isoleucine**	*ISO*	2.21	2.14
**Leucine**	*LEU*	3.91	3.78
**Lysine**	*LYS*	3.51	3.41
**Methionine**	*MET*	1.36	1.32
**Phenylalanine**	*PHE*	2.34	2.23
**Threonine**	*THRE*	2.10	2.05
**Valine**	*VAL*	2.41	2.31
***Non-essential Amino acids***			
**Alanine**	*Ala*	2.75	2.82
**Aspartic acid**	*Asp*	4.75	4.66
**Cystin**	*Cys*	0.63	0.59
**Glutamic acid**	*Glu*	9.77	9.65
**Glycine**	*Gly*	2.75	3.25
**Proline**	*Pro*	2.86	3.07
**Serine**	*Ser*	2.37	2.42
**Tyrosine**	*Tyr*	0.98	0.75
***Calculated values***			
**ΣΣAA(IAA+DAA)**		49.15	48.95
IAA/DAA**		0.83	0.80

*The IAA abbreviations are in capital letters and DAA in lowercase letters.

**IAA: Essential amino acids. DAA: Non-essential amino acids.

Apparent digestibility coefficients (ADCs) of amino acids (AAs) were measured at the end of the experiment using samples collected as described in [39]. Briefly, faeces were obtained from anaesthetized (Benzocaine, 25 μg kg^−1^) fish collected after a feed-deprivation period of 8 hours. Diets and faecal samples were analysed for dry matter, individual AA, and the digestibility marker (Yttrium, Merck KGaA, Darmstadt, Germany).

Diets and faeces were analysed for dry matter (DM, 105°C overnight). Yttrium was determined in diets and faeces using an atomic absorption spectrometer (Perkin Elmer 3300, Perkin Elmer, Boston, MA, USA) after nitric acid/hydrochloric acid digestion. Following the method described in [43], AA compositions of diets and faeces were determined using a Waters HPLC system (Waters 474, Waters, Milford, MA, USA) consisting of two pumps (Model 515, Waters), an auto sampler (Model 717, Waters), a fluorescence detector (Model 474, Waters), and a temperature control module. The amount of sample used was calculated to contain approximately 25 mg of crude protein that was hydrolysed with 50 mL of 6 N HCl with 0.5% phenol (v/v) at 115 °C for 24 h. Aminobutyric acid was added as an internal standard before hydrolysis. AAs were derivatised using 6-aminoquinolyl-N-hydroxysuccinimidyl carbamate (Waters, U.S.A.). Methionine and cysteine were determined separately as methionine sulphone and cysteic acid after oxidation with performic acid. AAs were separated by HPLC with a C-18 reverse-phase column Waters Acc. Tag (150 mm × 3.9 mm (Waters, U.S.A.).

The ADCs for individual AAs were calculated using the formula given in [40].

### Enzyme analysis

#### Sampling and preparation of enzyme extracts

At each sampling point (Table 1), 5 fish per tank were randomly collected to determine digestive enzyme activities. Fish sampling was always at the same time of the day and at least 2–3 hours after the last meal. Fish were dissected and the stomach and intestine separated. To obtain information about the proteolytic potential at the pH under which gastric digestion takes place, the buffer capacity of the diets was determined [44]. Measurements were made using a Crison pH25 pH-meter, equipped with a Crison 5208 microelectrode. The mEq/kg required to reach pH 3 is shown in the footnotes to Table 2. At the 2745 ddPSF sampling, the intestine was divided into 4 segments: pyloric caeca, anterior, mid- and posterior intestine (PC, AI, MI and PI). Dissection was conducted under a dissecting microscope on a pre-chilled glass plate maintained at 0 °C. Before removing gastrointestinal content, pH was measured in the stomach following [23] and in the intestine using a Crison 5208 microlectrode attached to a pH25 pH-meter. Samples were then flash-frozen in liquid nitrogen and stored at − 80°C until extraction.

Extracts were prepared by dilution of tissue samples in distilled water (1:10 w/v) and homogenization using the FastPrep-24^™^ Classic bead beating grinder and lysis system (MP Biomedicals, Solon, Ohio, USA) with the following conditions: 6.5 ms^-1^ and 20 s. After centrifugation at 12000 rpm for 15 min at 4°C, supernatants were collected and stored at −20°C until used for enzyme analysis. The concentration of soluble protein in extracts was determined by the Bradford method [45] using bovine serum albumin (2 mg ml^-1^) as standard.

#### Enzyme activities

Peptic activity was measured following the method described by Anson [46] and modified by [47] using hemoglobin 0.5% (w/v) as the substrate. Assays were carried out at pH 2.5 and at the pH determined in each stomach sampled. One unit of activity was defined as 1 μg of tyrosine released per min, and absorbance was measured at 280 nm.

Total alkaline protease activity (TAP) was measured using casein 1% (w/v) (Merck KGaA, Darmstadt, Germany) as the substrate [48]; one unit of activity was defined as 1 μg of tyrosine released per min, and absorbance was measured at 280 nm.

Trypsin and chymotrypsin activities were measured using 50 mM BApNA (N-α-benzoyl-Larginine-p-nitroanilide hydrochloride, Merck KGaA, Darmstadt, Germany) and 50 mM GApNA (N-glutaryl- L-phenylalanine-p-nitroanilide, Merck KGaA, Darmstadt, Germany) as substrates, respectively [49]. Absorbance at 405 nm was measured using a multiscan Ex spectrophotometer (Thermolab Systems, Helsinki, Finland). One unit of activity was defined as 1 μg of p-nitroaniline released per minute.

Alkaline phosphatase was analysed using p-nitrophenylphosphate (Merck KGaA, Darmstadt, Germany) as substrate (5 mM) in a solution of carbonate buffer (30 mM), pH 9, whereas aminopeptidase N activity was assayed using L-leucine p-nitroanilide (Merck KGaA, Darmstadt, Germany) (0.1 M) in a solution of phosphate buffer (80 mM), pH 7.0 as described in [50]. Enzyme activities were expressed as UA/g of fish (live weight) and were calculated as follows:

$$\frac{UA}{g\;fish}=\frac{\Delta Abs\;x\;OI}{\varepsilon \;x\;g\;tissue\;x\;t}\times dF$$

where UA = unit, *ΔAbs* is the absorbance increase at a determined wavelength; ε is molar extinction coefficient for the reaction product (mL μg^−1^ cm^−1^), *t* is the time of reaction and *dF* is the dilution factor *OI*, is the organ index the tissue mass (stomach or intestine) and fish weight ratio.

At the last sampling (2745 ddPSF), the specific activity (UA) was determined in four intestinal sections, and specific activities for enzyme activities in the entire intestine were calculated as follows:

$$\frac{UA}{g\;fish}=\frac{\left(PC\;\text{U}\;\text{x}\;\text{PC}\;\text{mass})\;+\;(\text{AI}\;\text{U}\;\text{x}\;\text{AI}\;\text{mass})\;+\;(\text{MI}\;\text{U}\;\text{x}\;\text{MI}\;\text{mass})\;+\;(\text{PI}\;\text{U}\;\text{x}\;\text{DI}\;\text{mass}\right)}{\text{Fish}\;\text{live}\;\text{weight}\;(\text{g})}$$

where UA = unit, PC = pyloric caeca, while AI, MI and PI stand for the anterior, mid- and posterior sections of the intestine, respectively.

### Statistical analysis

Data on intestinal fold height, width, thickness, numbers of mucus cells and numbers of folds in the AI and PI were analysed by two-way ANOVA with ploidy and diets as fixed factors and fish weight as covariate. Data on ADCs and enzyme activities were also analysed by two-way ANOVA with ploidy and diets as fixed factors. To analyse pepsin and gastric pH one-way ANOVA was carried out. Data normality and homogeneity of variances were checked using Shapiro-Wilk’s and Levene’s tests, respectively. When these conditions were not met, length measurements were logarithmically-transformed and count measurements were arcsin-transformed. If there were no differences between the replicates within each group, the data were pooled and analysed by one-way ANOVA. When there were differences among replicates within one or several groups, the groups were compared by nested ANOVA using tanks nested in the dietary group. In the absence of normality, data were analysed by Kruskal-Wallis test. In cases of significant differences among groups, pairwise comparisons were made using Tukey’s or Gabriel Post-hoc tests. In addition, a statistical evaluation of the data from enzyme activities was carried out using a 4 x 2 x 2 factorial arrangement of treatments in a completely randomised experimental design (three-way ANOVA), with age (sampling time), diet, and ploidy as fixed factors. All data were recorded and processed using SigmaPlot v.14.0 and the statistical analyses performed using SYSTAT v.13 (SYSTAT Software Inc., USA). The level of significance was *P*<0.05, and the results are presented as means ± Standard Deviation (SD) or Standard Error (SE).

## Results

### Histomorphology at hatch

No obvious differences could be observed in the digestive tract histomorphology of newly-hatched diploid and triploid fish (S1 Fig). Stratified squamous epithelium lined the buccopharynx and scattered mucus cells and taste buds were present. The esophagus was short, with scattered mucus cells in the epithelium. No clear transitional zone between the esophagus and the stomach was seen. The incipient stomach was a straight, sac-like structure with longitudinal mucosal folds. Connective tissue was dominant in the folds (lamina propria) and columnar epithelium was facing the lumen. Neither gastric glands nor pyloric caeca were present. The intestine was a simple straight tube without coiling. In the anterior part, mucosal folding was noted while the posterior part was lined with simple columnar epithelium without any sign of folding and no mucus cells were present. The rectum was short and the anal opening was present. The liver appeared functional with vacuolated hepatocytes (lipid globules) and the pancreas had zymogen granules in the exocrine part.

#### Histology at start-feeding (0 ddPSF)

**Fig 3:**
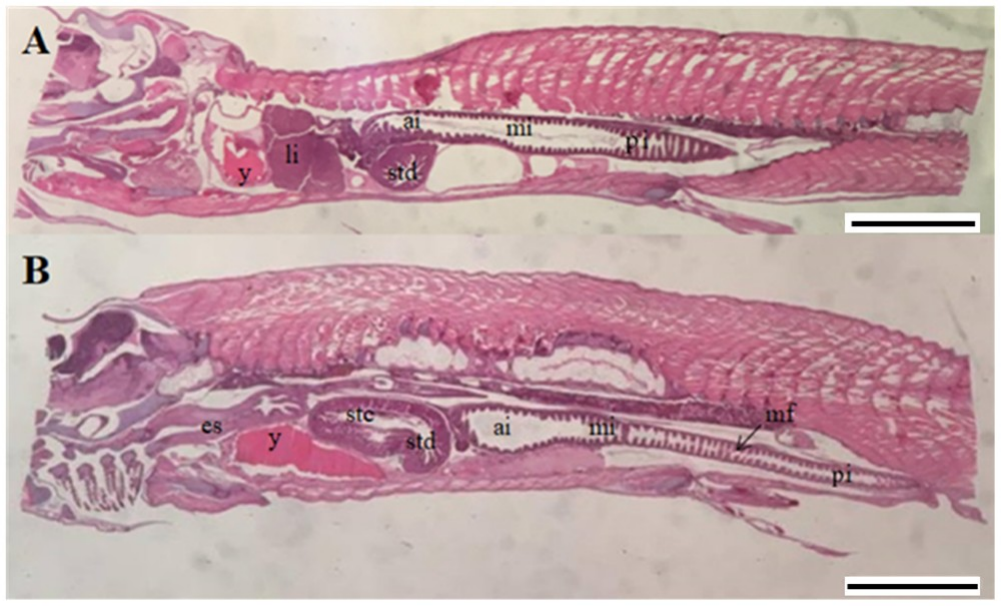
Longitudinal sections of the abdominal part of whole individuals of diploid and triploid Atlantic salmon at start-feeding (0 ddPSF). (A) Overview whole individual (2n). (B) Overview whole individual (3n). ai. anterior intestine; es. esophagus; li. liver; mf. mucosal folds; mi. mid intestine pi. posterior intestine; stc. stomach cardiac; std. stomach posterior; y. yolk sac. Scale. A-B. 1 mm.

**Fig 4:**
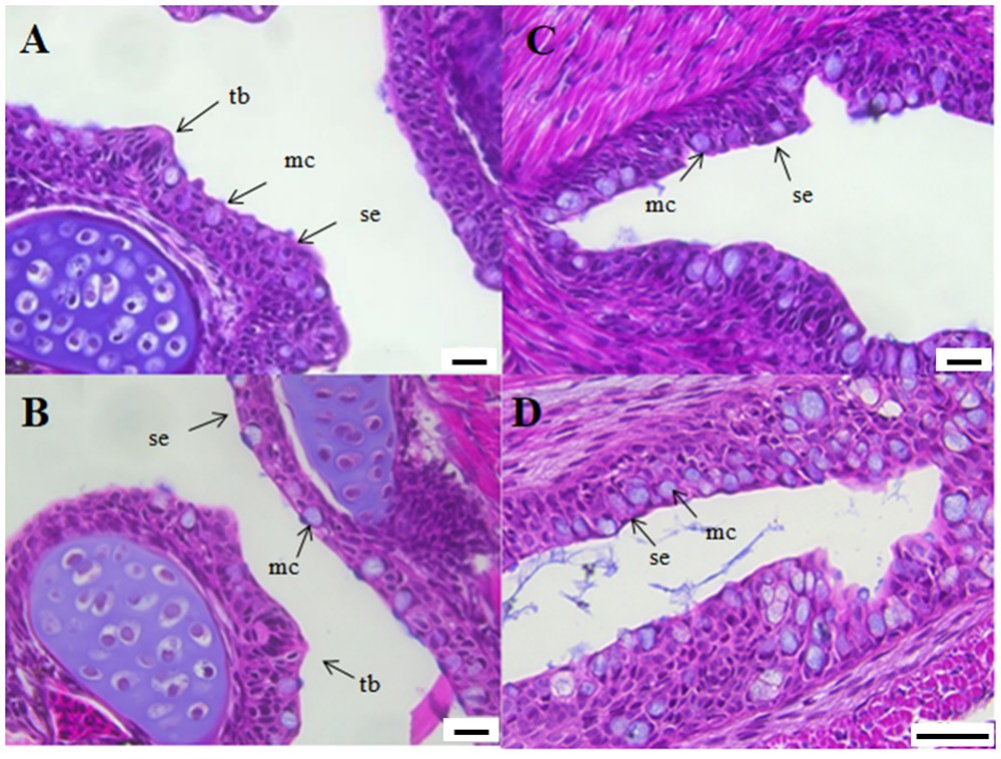
Longitudinal sections of the pharynx (A-B) and esophagus (C-D) of diploid and triploid Atlantic salmon at start-feeding (0 ddPSF. (A) Diploid individual (2n). (B) Triploid individual (3n). (C) Diploid individual (2n). (D) Triploid individual (3n). mc. mucus cells; se. squamous epithelium; tb. taste buds. Scale. A-C. 20 μm. D. 50 μm.

At start-feeding yolk remains were still present in the abdominal cavity (Fig 3) and no obvious histological differences between the ploidies were registered. A stratified squamous epithelium lined the bucco-pharyngeal cavity and mucus cells and taste buds were numerous (Fig 4A and 4B). The epithelium of the esophagus had started to fold, and was characterized by numerous mucus cells (Fig 4C and 4D).

**Fig 5:**
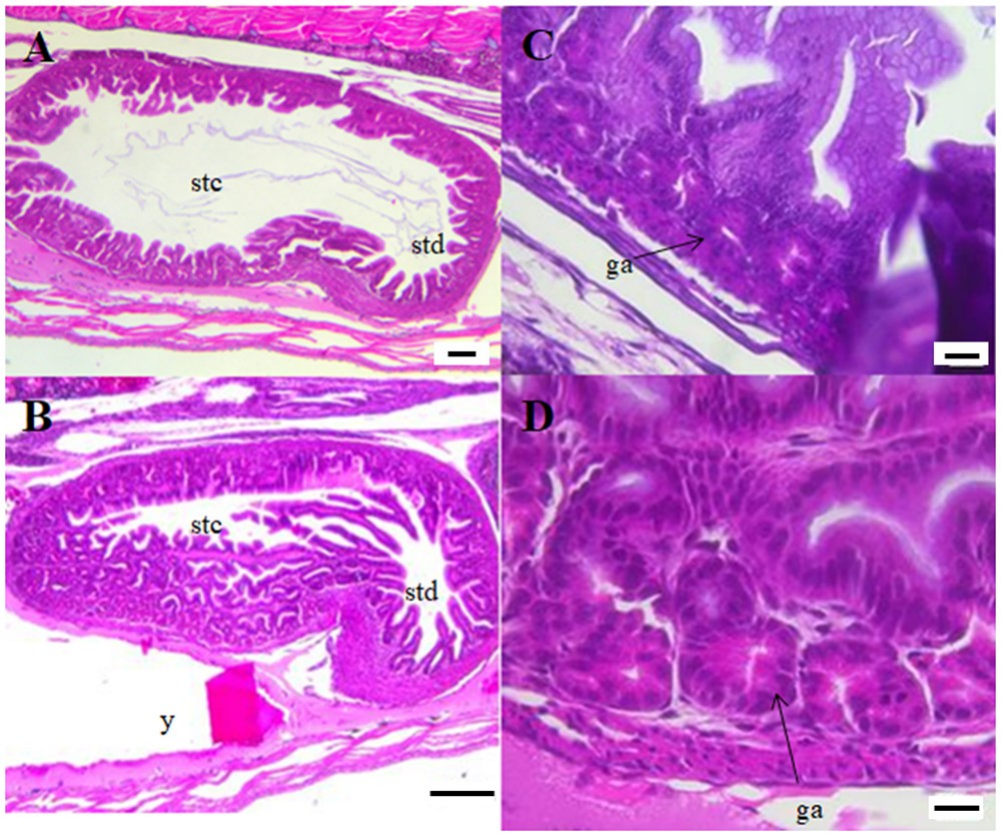
Longitudinal sections of the stomach (A-B) and gastric glands (C-D) of diploid and triploid Atlantic salmon at start-feeding (0 ddPSF). (A) Diploid individual (2n). (B) Triploid individual (3n). (C) Gastric glands in the cardiac part of the stomach (2n). (D) Gastric glands in the cardiac part of the stomach (3n). ga. gastric pits; stc. stomach cardiac; std. stomach posterior; y. yolk sac. Scale. A. 100 μm. B. 200 μm. C-D. 20 μm.

The stomach was J-shaped (Fig 5A and 5B) and the mucosa of the cardiac part was lined with simple secretory columnar cells with tubular gastric glands (Fig 5C and 5D). The posterior part had a thicker muscle layer and more folded mucosa. Mucus cells were present in the epithelium along the full length of the stomach.

**Fig 6:**
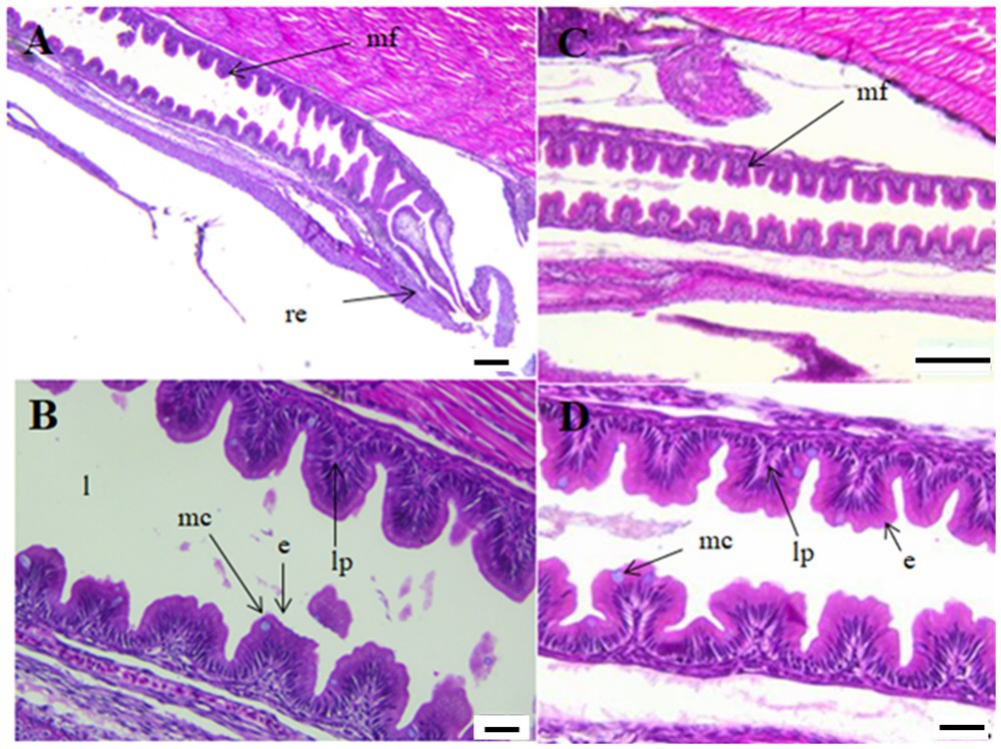
Longitudinal sections of posterior intestine of diploid and triploid Atlantic salmon at start-feeding (0 ddPSF). (A) Posterior intestine (2n). (B) Detail of posterior intestine (2n). (C) Posterior intestine (3n). (D) Detail of posterior intestine (3n). e. enterocytes; l. lumen; lp. lamina propria; mc. mucus cells; mf. mucosal folds; re. rectum. Scale. A,C. 200 μm. B,D. 50 μm.

The intestine was slightly coiled, and characterized by a simple folded mucosa (Fig 6) with a few mucus cells. No supranuclear vacuoles were present. The liver showed variable vacuolization of the hepatocytes within both ploidies. Pancreatic tissue was present in the mesenteric tissue of the abdomen, particularly around the pyloric caeca and anterior intestine, and zymogen granules were prominent.

#### Histology and morphometry after start-feeding (218–2745 ddPSF)

**Fig 7:**
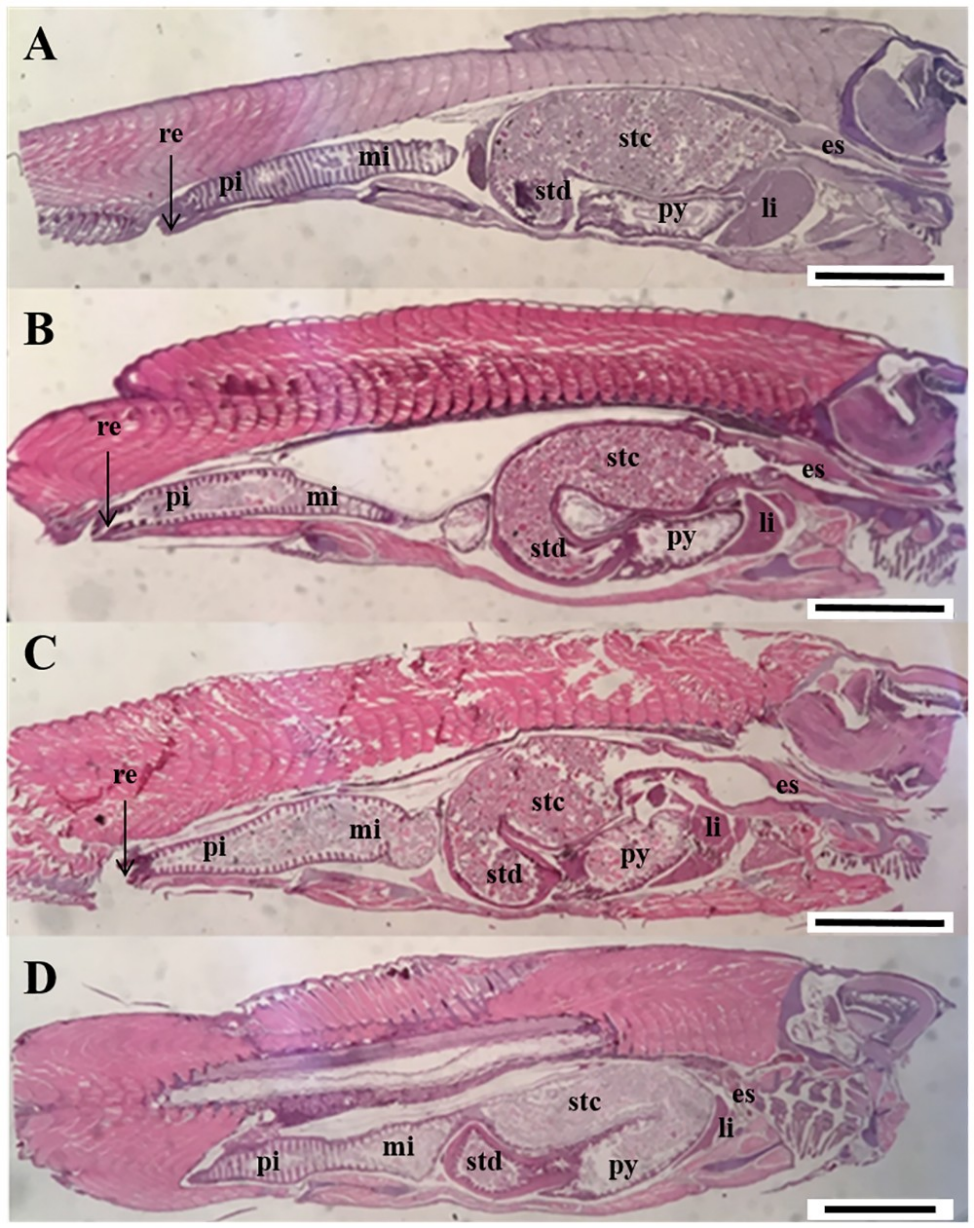
Longitudinal sections of the abdominal part of whole individuals of diploid and triploid Atlantic salmon fed different diets at 218 ddPSF. (A) 2nSTD, (B) 2nHFM, (C) 3nSTD, (D) 3nHFM). es. esophagus; li. liver; mi. mid intestine; pi. posterior intestine; py. pyloric caeca; re. rectum; stc. stomach cardiac; std. stomach posterior. Scale. A-D. 1.88 mm.

**Fig 8:**
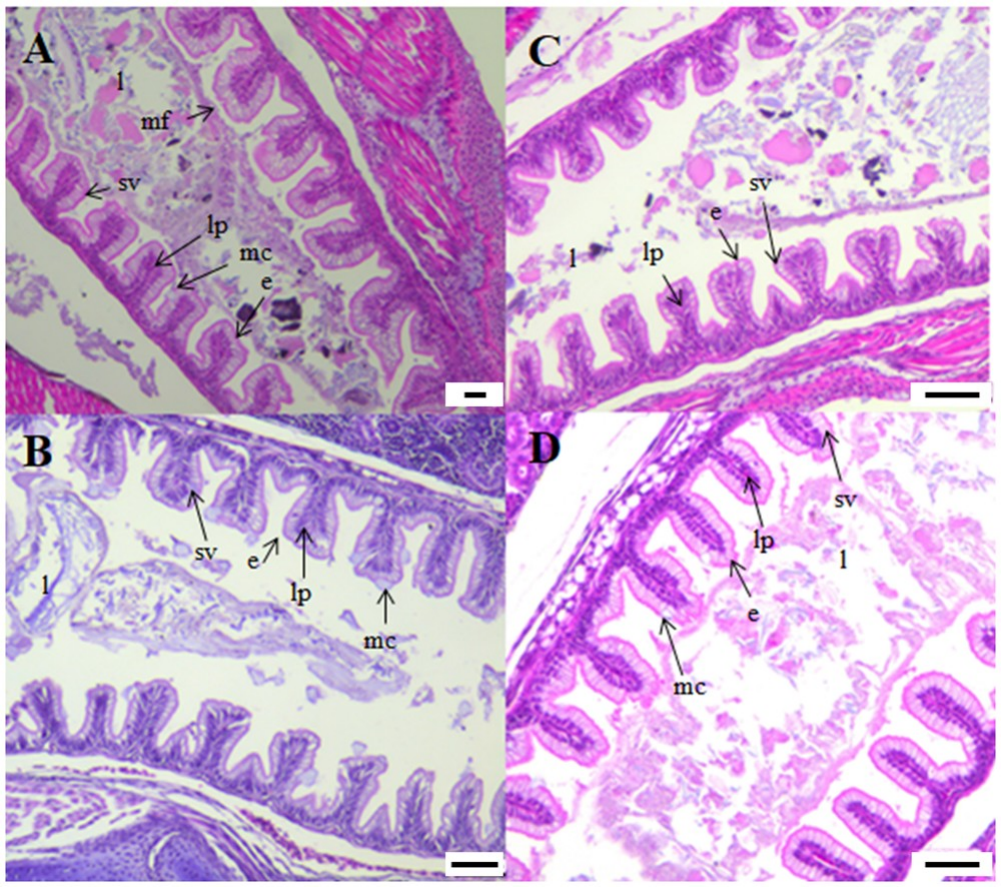
Longitudinal sections of the posterior intestine of diploid and triploid Atlantic salmon at 218 ddPSF. (A) 2nSTD, (B) 2nHFM, (C) 3nSTD, (D) 3nHFM). e. enterocytes; l. lumen; lp. lamina propria; mc. mucus cells; mf. mucosal folds; sv. supranuclear vacuoles. Scale. A. 50 μm. B-D. 100 μm.

No obvious differences among groups could be observed in the digestive tract histomorphology at 218 ddPSF (Fig 7). Individual differences with regard to liver vacuolization within dietary groups and ploidies were noted. The yolk sac was completely absorbed and in comparison with earlier samples there was an increase in numbers and sizes of mucus cells in the pharynx and increased mucosal folding and more numerous taste buds and mucus cells in the esophagus. There was a clear transitional zone between the esophagus and stomach (Fig 7). The cardiac and posterior stomach had developed an extended J-shape and loop (Fig 7) with a thicker muscle layer than recorded earlier. Pyloric caeca appeared longer and more numerous than previously and there was an increase in mucosal fold height. A general increase in size of the intestine as well as fold height and width were noted. Small supranuclear vacuoles were observed in the enterocytes (Fig 8). The hepatocytes had numerous glycogen granules and variable degrees of vacuolization. Pancreatic tissue had expanded and histomorphology appeared similar between groups.

An apparent increase in both number and size of mucus cells was noted in the pharyngeal mucosa between 218 and 580 ddPSF while no major differences were registered in the oesophageal mucosa. The stomach mucosa showed a general increase in muscle layer thickness. There was a distinct separation between the cardiac and posterior stomach, and pyloric caeca were numerous. The intestinal fold heights and muscle layers had increased and small supranuclear vacuoles characterized the enterocytes. The liver was relatively large and vacuolated in all samples and no major modifications were registered in the pancreatic tissue.

Morphometric measurements of the intestine revealed only minor differences among ploidy and dietary groups and these were mostly related to the anterior section (Table 4). At 1455 ddPSF, differences were observed in thickness of the anterior intestinal wall with 3nSTD fish having a significantly thinner intestinal wall (19.60 μm ± 1.09) than 3nHFM fish (25.09 μm ± 1.09) (P<0.01) and 2nHFM fish (25.65 ± 1.74) (P<0.01) but not diploid siblings fed the STD diet.

**Table 4:** Morphometric measurements of anterior and posterior sections of the intestine in diploid and triploid Atlantic salmon at 1455–2745 ddPSF. Measurements refer to intestinal fold height and width, thickness of the intestinal wall, number of mucus cells and folds in the two sections of the intestine.

		Anterior intestine				
Age (ddPSF)	Group	Height (μm)	Width (μm)	Thickness (μm)	Mucus cells (nr)	Folds (nr)
***1455***	2nSTD	168.70±3.85	99.04±3.66	23.43±1.68^a.b^	3.54±0.06	5.76±0.15
	2nHFM	151.35±13.30	82.35±5.49	25.65±1.74^b^	3.29±0.16	6.03±0.20
	3nSTD	149.85±13.64	89.53±10.68	19.64±1.09^a^	3.16±0.08	5.63±0.18
	3nHFM	179.98±16.57	108.91±8.52	25.09±1.09^b^	3.47±0.09	5.80±0.16
***2090***	2nSTD	195.361±8.20	93.17±3.76^a.b^	47.37±3.77^a.b^	3.69±0.14	6.64±0.15
	2nHFM	168.69±10.00	79.65±4.48^a^	36.13±1.53^a^	3.25±0.10	6.49±0.26
	3nSTD	209.85±15.01	97.32±4.18^b.c^	56.75±3.98^b^	3.46±0.11	6.90±0.18
	3nHFM	201.85±16.45	111.13±2.58^c^	35.93±2.32^a^	3.39±0.08	6.36±0.19
***2745***	2nSTD	184.06±10.12	102.04±6.56	61.76±4.83^a.b^	3.43±0.11	6.97±0.06
	2nHFM	216.58±15.04	104.58±7.39	54.47±4.09^a^	3.41±0.19	6.97±0.13
	3nSTD	219.24±17.41	105.68±4.49	93.02±10.34^b^	3.45±0.14	7.24±0.19
	3nHFM	217.33±9.53	107.65±4.94	56.36±3.22^a^	3.24±0.07	6.93±0.16
		**Posterior intestine**					
***1455***	2nSTD	243.26±18.86	125.14±6.42	20.84±1.38	3.29±0.08	6.24±0.17
	2nHFM	318.56±19.49	109.94±4.86	21.61±2.03	3.16±0.19	6.19±0.22
	3nSTD	319.80±25.16	126.33±5.65	22.55±1.49	3.28±0.12	6.30±0.10
	3nHFM	290.71±23.12	132.45±5.89	19.07±1.50	3.28±0.16	6.11±0.17
***2090***	2nSTD	415.99±27.15	126.79±8.03	37.60±3.75	3.50±0.15	7.70±0.24
	2nHFM	398.22±14.57	136.97±4.83	31.39±2.08	3.46±0.08	7.36±0.09
	3nSTD	383.67±15.76	133.63±8.07	35.92±2.33	3.51±0.08	7.35±0.09
	3nHFM	410.71±7.33	146.91±4.92	31.31±2.09	3.41±0.11	7.19±0.12
***2745***	2nSTD	354.26±15.05^a.b^	139.89±7.52	37.28±2.69	3.21±0.09	7.73±0.22
	2nHFM	432.46±7.16^b^	135.12±8.33	37.99±2.19	3.25±0.15	8.10±0.31
	3nSTD	329.06±34.80^a^	121.68±7.48	40.13±1.12	3.10±0.11	7.40±0.25
	3nHFM	362.95±25.46^a.b^	148.11±8.01	36.90±3.46	3.25±0.11	7.27±0.16

For each sampling point, data in the same column with different superscripts differ at P<0.05 (two-way ANOVA). Data reported as mean ± standard error (SE).

At 2090 ddPSF, the condition in the anterior intestine was reversed and 3nSTD fish had a significantly thicker intestinal wall (56.75 μm ± 3.98) than 3nHFM fish (35.93 μm ± 2.32) (P<0.05) and 2nHFM fish (36.13 μm ± 1.53) (P<0.01) (Table 4). At the same time, the width of folds in the anterior intestine was generally greater in triploids than in diploids, with 3nHFM fish (111.13 ± 2.58) having wider folds than 2nHFM fish (79.65 ± 4.48) (P<0.001) and 2nSTD fish (93.17 ± 3.76) (Table 4).

At 2745 ddPSF, differences recorded in the anterior intestine between treatment groups were in line with observations made at the previous sampling point with 3nSTD fish having a significantly thicker intestinal wall (93.02 μm ± 10.34) than 3nHFM fish (56,36 μm ± 3.22) (P<0.05) and 2nHFM fish (79.6 μm ± 4.48) (P<0.001). In the posterior intestine, differences were only observed in fold size where 2nHFM fish had significantly longer folds (432.46 μm ± 7.16) than 3nSTD fish (329.06 μm ± 34.80) (P<0.001) (Table 4).

### Enzyme activity (875–2745 ddPSF)

**Fig 9:**
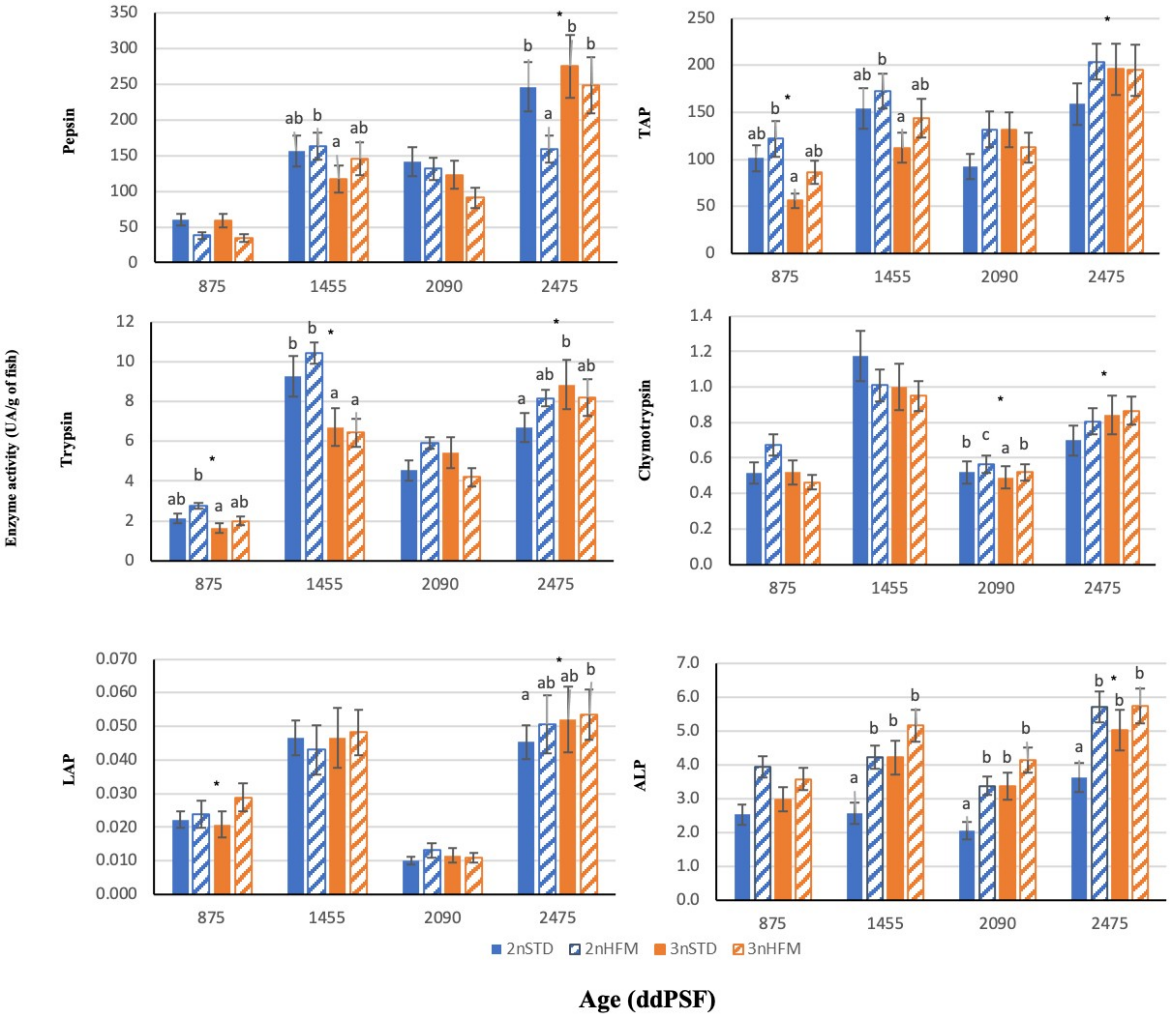
Digestive enzymes activities of diploid (2n) and triploid (3n) Atlantic salmon, *Salmo salar*, fed fish meal (STD) and hydrolysed fish protein (HFM) diets. Pepsin, total alkaline proteases (TAP), trypsin, leucine aminopeptidase (LAP), chymotrypsin and alkaline phosphatase (ALP) activities are expressed as UA/g of fish measured at 875, 1455, 2090 and 2745 ddPSF (see Table 1). Different lower case letters denote significant differences (P<0.05) between groups. (*) Indicates significant differences between 2n and 3n in each sampling (P<0.05). Data are presented as means ± SE (n = 15).

There were significant effects of feed type (HFM, STD), and age on specific activity of gastric pepsin, but there were significant interactions that may have masked major trends (S1 Table). Specific activity of pepsin in the stomach tended to increase over time, although there was a transient dip at 2090 ddPSF during the short daylight regime used to simulate winter conditions and induce parr-smolt transformation (S1 Table, Fig 9). Differences between groups were detected in peptic activity at 1455 ddPSF, being lower in triploid fish fed STD diet than diploid fish fed HFM diet, but at the last sampling the situation was reversed (Fig 9).

Considering diploids, at the last sampling point (2745 ddPSF) peptic activity was higher (*P*<0.05) in fish fed the STD diet than in those fed the HFM diet; this was a general trend for all sampling points, but significant differences were not observed other than at the final sampling (Fig 9). For triploids, no clear pattern emerged and peptic activity was not significantly different at any sampling point (Fig 9).

Peptic activity at 2745 ddPSF was assayed at both pH 2.5 and the pH measured in the stomach during fish sampling (Table 5). Peptic activity at *in situ* gastric pH was lower than the activity measured at pH 2.5 (Table 5). The buffering capacity of the HFM diet was lower than that of the STD diet (Table 2), and fish of the 3nHFM group had the lowest measured gastric pH (3.7 ± 0.289, *P<0*.*05*). When pepsin was determined at *in situ* gastric pH, fish from the 3nHFM group exhibited the highest activity probably as a result of the low pH (Table 5).

**Table 5:** Gastric pH (±SE) at 2745 ddPSF sampling in the four experimental fish groups and peptic activity (UA/g of fish) measured at *in situ* gastric pH and at pH 2.5.

		Peptic activity (UA/ g fish)	
	Gastric pH	*in situ* pH	pH 2.5
**2nSTD**	5.04^b^ ±0.278	128^b^ ±12.4	218^b^ ±21.0
**2nHFM**	5.10^b^ ±0.232	78^a^ ±10.8	133^a^ ±18.4
**3nSTD**	4.84^b^ ±0.251	168^bc^ ±11.1	248^b^ ±16.3
**3nHFM**	3.69^a^ ±0.289	198^c^ ±18.4	222^b^ ±20.7

Data in the same column with different superscripts differ at P<0.05. SE: standard error of the mean.

Intestinal enzyme activities were characterized by relatively large fluctuations (Fig 9), and there was a significant effect of age on the specific activity of each enzyme assessed (S1–S6 Tables).

### Hydrolysed

**Fig 10:**
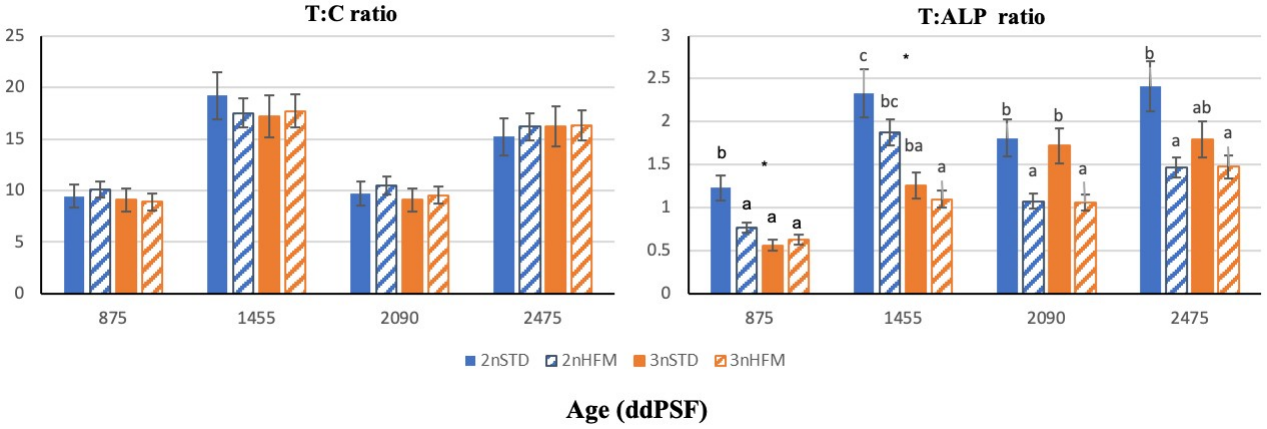
Trypsin and chymotrypsin ratio (T:C) and trypsin and ALP ratio (T:ALP) of diploid (2n) and triploid (3n) Atlantic salmon, *Salmo salar*, fed fish meal (STD) and hydrolysed fish protein (HFM) diets at 875, 1455, 2090 and 2745 ddPSF sampling (see Table 1). Data are presented as means ± SE (n = 15). Different lower case letters denote significant differences (P<0.05) between groups. (*) Indicates significant differences between 2n and 3n in each sampling (P<0.05). Data are presented as means ± SE (n = 15).

In general, lowest intestinal enzyme activities were registered at 875 ddPSF and low activities were also noted for most enzymes at 2090 ddPSF, the latter sampling time being during the short daylight regime used to simulate winter conditions and induce parr-smolt transformation. The fluctuation in the level of enzyme activities and marked drop at 2090 ddPSF was most evident for LAP (Fig 9). There were low ratios of trypsin to chymotrypsin (T:C ratio) at 875 and 2090 ddPSF but this was not observed for the trypsin to ALP ratio at 2090 ddPSF (Fig 10).

With the exception of the 2090 and 2745 ddPSFsampling points, diploid fish had higher trypsin activities than triploid fish, with triploids fed STD exhibiting higher trypsin activity than diploids fed STD at 2745 ddPSF. Likewise, total alkaline protease activity (TAP) was lower in diploids fed STD than triploids at 2745 ddPSF (Fig 9). For brush border enzymes, LAP activity was higher in triploids than diploids at 875 and 2745 ddPSF (Fig 9), and triploids had higher ALP activity than diploids fed STD diet at 1455 and 2745 ddPSF. There were no significant differences in T:C ratios between ploidies or diets at any sampling time (Fig 10). By contrast, diploids fish fed STD exhibited higher T:LAP ratio than triploid fish fed HFM diet in all samplings, and than diploids fed HFM at 875, 2090 and 2475 ddPSF. T:ALP was higher in diploids than triploids at 875 and 1455 ddPSF (Fig 10).

**Fig 11:**
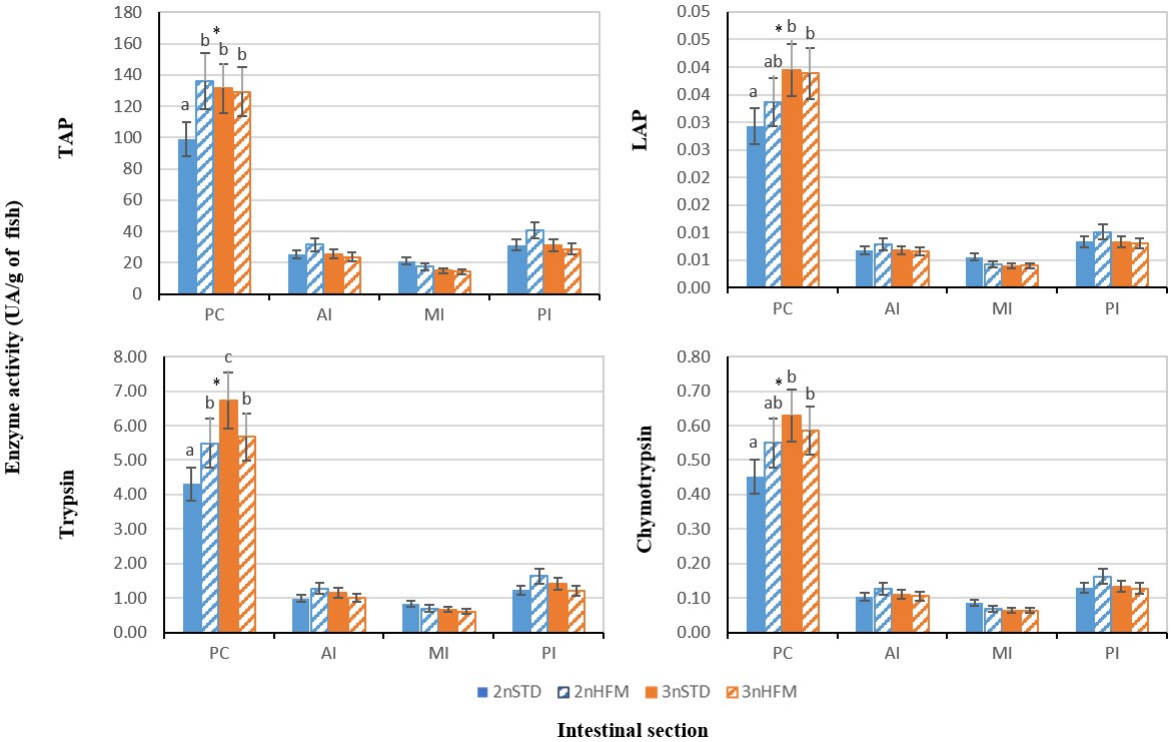
Total alkaline proteases (TAP), trypsin, leucine aminopeptidase (LAP) and chymotrypsin activity (expressed as UA/g of fish) of diploid (2n) and triploid (3n) Atlantic salmon, *Salmo salar*, fed fish meal (STD) and hydrolysed fish protein (HFM) diets measured at 2745 ddPSF sampling point (see Table 1: 38 weeks after start feeding). Four intestinal sections were considered: pyloric caeca (PC), anterior intestine (AI), medium intestine (MI) and posterior intestine (PI). Different letters denote significant differences (P<0.05). Data are presented as means ± SE (n = 15). (*) Indicates significant differences between 2n and 3n in each section (P<0.05).

**Fig 12:**
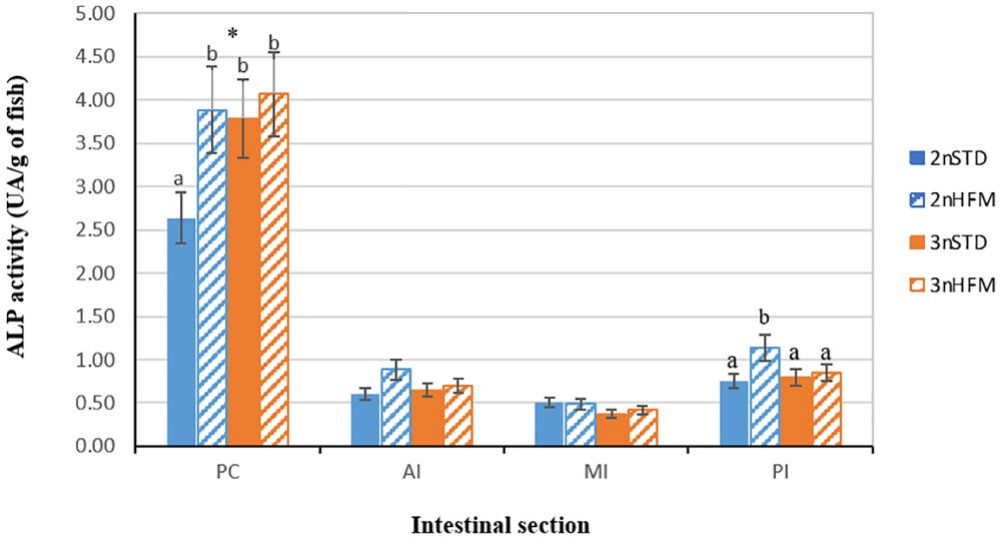
Alkaline phosphatase (ALP) activity (expressed as UA/g of fish) of diploid (2n) and triploid (3n) Atlantic salmon, *Salmo salar*, fed fish meal (STD) and hydrolysed fish protein (HFM) diets measured at 2745 ddPSF sampling point (see Table 1: 38 weeks after start feeding). Four intestinal sections were considered: pyloric caeca (PC), anterior intestine (AI), medium intestine (MI) and posterior intestine (PI). Different letters denote significant differences (P<0.05). Data are presented as means ± SE (n = 15). (*) Indicates significant differences between 2n and 3n in each section (P<0.05).

At 2745 ddPSF four sections of intestine were taken with the aim to study enzyme activity along its length; pyloric caeca (PC), anterior intestine (AI), mid-intestine (MI) and posterior intestine (PI). Overall, the pyloric caeca showed the highest activities for all enzymes evaluated (Fig 11). With regard to ploidy and intestinal section triploids fed STD diet exhibited higher TAP, chymotrypsin and LAP activity than diploids fed the same diet in the PC (Fig 11). Diploid fish fed STD presented the lowest ALP activity in the PC section and lower than diploid fish fed HFM in PI section (Fig 12).

### Relationships between enzyme activity and TGC

**Fig 13:**
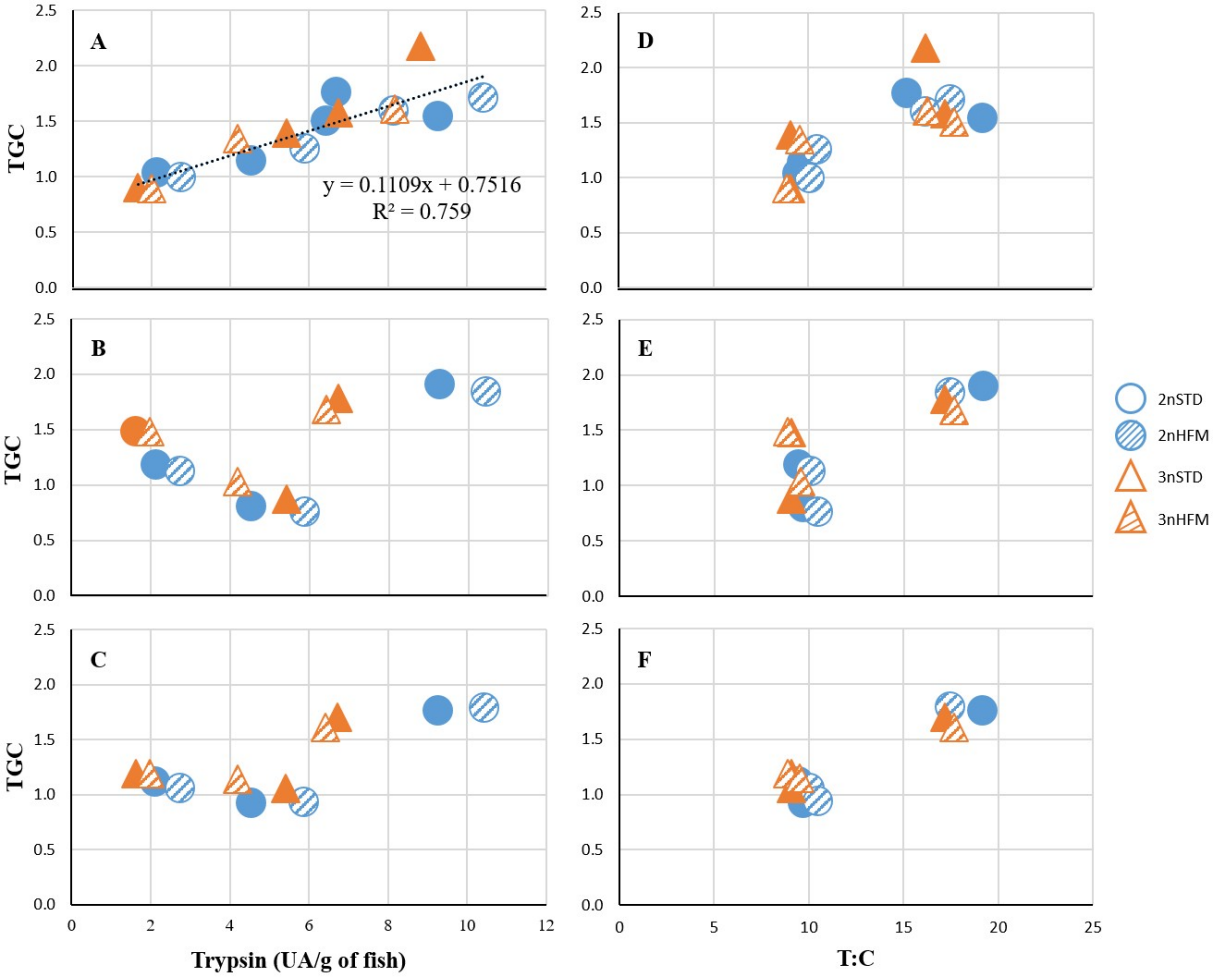
Relationship between TGC and trypsin activity or trypsin-to-chymotrypsin ratio (T:C). TGC data and specific activity of trypsin (A-C) activity (expressed as UA/g of fish) and trypsin-to-chymotrypsin (T:C) ratio (D-F) recorded in the intestine of diploid and triploid fish fed fish meal (STD) and hydrolysed fish protein (HFM) diets in the period (A,D) prior to sampling, (B,E) after sampling and (C,F) in whole timeframe.

There was a significant positive relationship between TGC in the period prior to sampling and the specific activity of trypsin recorded in the intestine (Fig 13A). hydrolysedOn the other hand, the relationship was not significant when either the growth period subsequent to sampling or the period that spanned both preceding and subsequent growth was considered (Fig 13B and 13C). The T:C activity ratios were low at 2090 ddPSF (Fig 10), and this coincided with a period of poor growth of the fish during the time of manipulation of photoperiod to induce parr-smolt transformation. Numerical values for T:C were very similar for both ploidies over time, and at a given sampling point differences between fish given the STD and HFM diets were minimal. When the T:C data for the different sampling points, ploidies and feed types were combined with TGCs for periods prior to and after sampling and for the periods that spanned sampling points the data formed two distinct clusters. Low T:C values coincided with times of low rates of growth (low values for TGC), and vice versa (Fig 13D–13F).

**Fig 14:**
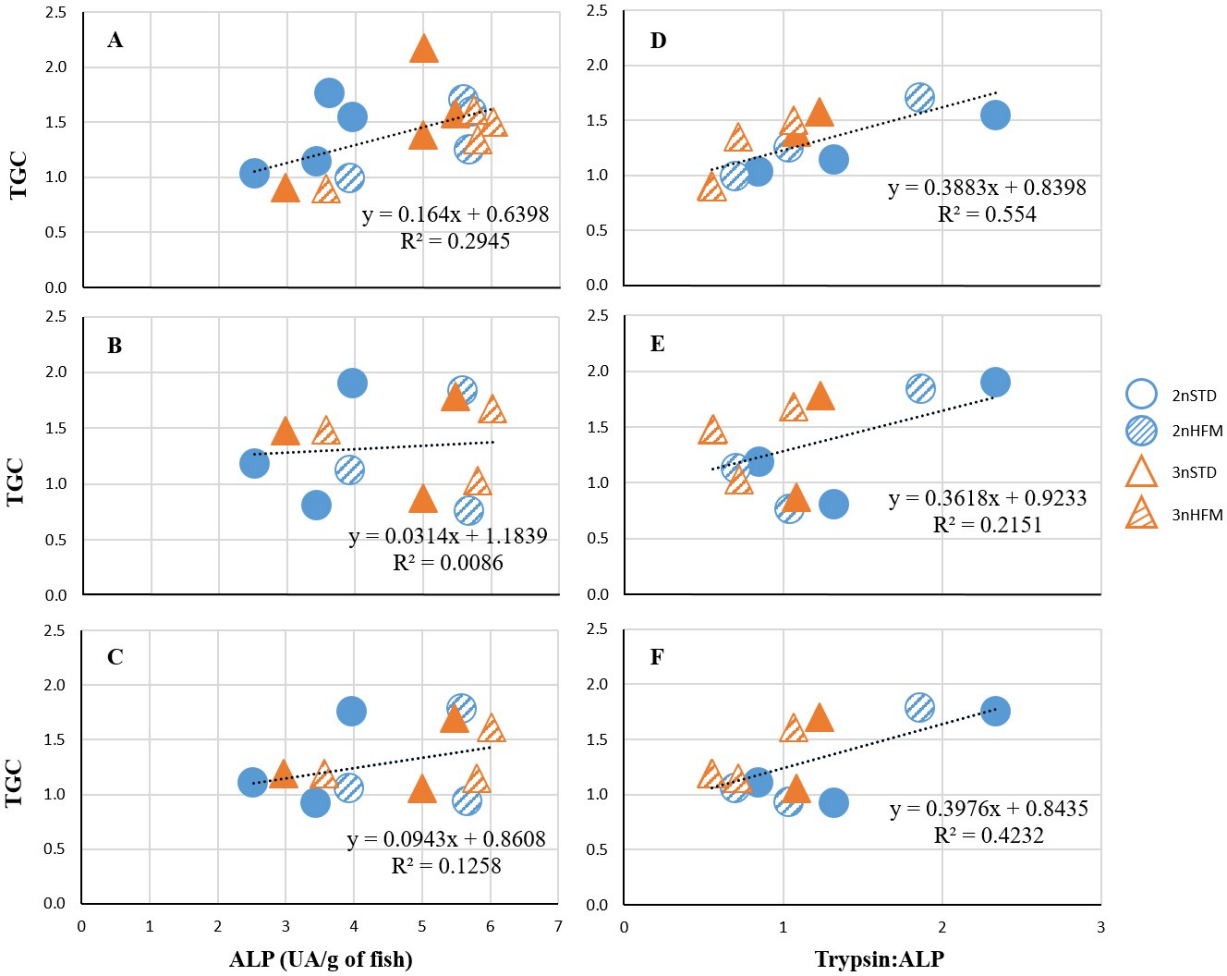
Relationship between TGC and ALP activity or trypsin-to-ALP ratio. TGC data and specific activity of ALP (A-C) activity (expressed as UA/g of fish) and trypsin-to-ALP ratio (D-F) recorded in the intestine of diploid and triploid fish fed fish meal (STD) and hydrolysed fish protein (HFM) diets in the period (A,D) prior to sampling, (B,E) after sampling and (C,F) in whole timeframe.

There were significant, but weak, positive relationships between TGC and ALP activities for growth registered in the periods prior to, after and spanning the time at which intestinal samples were taken for analysis (Fig 14A–14C). A positive relationship was observed between TGC and relative activities of luminal and brush-border enzymes (trypsin-to-ALP ratios) for all periods (Fig 14D–14F).

### AA digestibility

A ploidy effect was observed with regard to AA digestibility (Table 6), where diploid fish had higher ADCs for every AA (P<0.01). Within ploidies, there were no consistent significant dietary effects on AA digestibility and no significant interaction (ploidy and diet) was observed.

**Table 6:** Apparent digestibility coefficients of individual AAs in each diet and ploidy group.

	Group				Two-way ANOVA		
	2nSTD	2nHFM	3nSTD	3nHFM	Diet	Ploidy	Interaction
**ARG**	95.0^b^ ±0.13	95.0^b^ ±0.36	94.3^a^ ±0.17	93.7^a^ ±0.55	ns	**	ns
**HIS**	91.7^b^ ±0.48	91.0^b^ ±0.67	89.7^a^ ±0.93	89.8^a^ ±0.56	ns	**	ns
**ISO**	92.2^b^ ±0.30	91.8^ab^ ±0.73	90.7^ab^ ±1.30	90.1^a^ ±0.13	ns	**	ns
**LEU**	93.2^b^ ±0.26	92.8^b^ ±0.71	91.9^ab^ ±0.87	91.4^a^ ±0.19	ns	**	ns
**LYS**	93.3^b^ ±0.26	93.0^b^ ±0.67	91.8^a^ ±0.68	91.5^a^ ±0.25	ns	**	ns
**MET**	93.1^c^ ±0.27	92.4^bc^ ±0.07	91.3^ab^ ±1.19	90.2^a^ ±0.43	ns	**	ns
**PHE**	93.1^b^ ±0.27	92.8^b^ ±0.68	91.5^a^ ±0.83	91.3^a^ ±0.18	ns	**	ns
**THR**	89.8 ±0.11	89.5 ±1.25	87.5 ±1.49	87.7 ±0.50	ns	**	ns
**VAL**	91.5^b^ ±0.33	91.1^b^ ±0.72	89.6^a^ ±0.92	89.3^a^ ±0.04	ns	**	ns
**Ala**	92.4^b^ ±0.24	92.2^b^ ±0.61	90.2^a^ ±0.84	90.2^a^ ±0.41	ns	*	ns
**Asp**	83.1^b^ ±0.48	83.1^b^ ±1.58	79.7^a^ ±1.69	80.1^a^ ±0.52	ns	**	ns
**Cys**	82.5^b^ ±0.67	81.3^b^ ±1.47	78.2^a^ ±2.71	78.0^a^ ±0.96	ns	**	ns
**Glu**	95.3^c^ ±0.18	94.5^b^ ±0.49	94.0^ab^ ±0.58	93.3^a^ ±0.25	*	**	ns
**Gly**	87.8^b^ ±0.34	88.7^b^ ±1.04	83.4^a^ ±1.84	85.5^a^ ±0.88	ns	**	ns
**Pro**	93.8^b^ ±0.23	93.0^b^ ±0.49	91.3^a^ ±0.78	91.0^a^ ±0.38	ns	**	ns
**Ser**	91.1^b^ ±0.28	90.7^b^ ±0.37	88.9^a^ ±1.32	88.8^a^ ±0.39	ns	**	ns
**Tyr**	88.7 ±0.43	89.3 ±1.02	87.3 ±1.64	87.4 ±0.77	ns	**	ns

Apparent digestibility coefficients (ADCs) for individual AAs in diploid (2n) and triploid (3n) Atlantic salmon, *Salmo salar*, fed fish meal (STD) and hydrolysed fish protein (HFM) diets. The IAA abbreviations are in capital letters and DAA in lowercase letters. Values presented as mean ± SD (n = 3). Different superscript letters in each row denote significant differences. Two-way ANOVA: ns: non-significant (P ≥ 0.05);

*P < 0.05;

**P < 0.01; Two-way ANOVA revealed no interaction (Diet x Ploidy).

## Discussion

The histology of the digestive system of the newly-hatched and start-feeding diploid and triploid Atlantic salmon did not differ substantially, and reflected organ maturation and functionality. Macroscopically, the digestive system of the Atlantic salmon corresponds to the general form seen in other salmonids and several other teleosts [51–53]. The stomach is J-shaped, with an enlarged lumen, and the intestine is comprised of the upper (anterior or proximal) and lower posterior intestine that differ in structure and histology. The proximal intestine has several blind-ended diverticula, the pyloric caeca, annexed to its anterior part immediately adjacent to the pyloric sphincter. The posterior intestine is characterized by having a larger diameter than the proximal intestine, pronounced folds that protrude towards the lumen, a thinner muscularis layer and fewer goblet cells than seen in the proximal intestine. Morphometric analysis of the intestine of the older fish (1455–2745 ddPSF) examined in our study revealed that there were changes and variations over time, but without consistent differences between ploidies and feed types (Table 4). Unlike the situation in Atlantic cod, *Gadus morhua*, in which triploids have higher concentrations of mucus cells in the intestinal folds than diploids [54], there were no differences between the ploidies of the Atlantic salmon examined in our study. Fish fed the STD diet tended to have thicker intestinal walls, both in the anterior and posterior intestine, and the same trend was seen in intestinal fold numbers. Irrespective of diet and ploidy, numbers of folds were greater in the posterior, than in the anterior intestine, and the wall of the posterior intestine was thinner than that of the anterior intestine (Table 4). These observations, which conform to previous findings for salmonids [27,55], are thought to reflect a more vigorous and pronounced peristaltic activity in the anterior part of the intestine compared to the posterior [28]. Although we did not observe consistent differences between diploids and triploids in intestinal histology, there are reports of ploidy differences in gut morphology, with triploids having shorter intestines and fewer pyloric caeca than diploids [6]. These differences could have an influence on the surface area available for nutrient absorption, and result in differences in nutrient absorption efficiencies between diploids and triploids.

In addition to the area available for nutrient absorption, enzyme activities are expected to influence the efficiency with which nutrients are digested and absorbed, and be linked to feed utilization and growth. For example, there is some evidence for a link between protease activity and growth in salmonids and some other fish species [25,56–60]. In our study, the low enzyme activities recorded at 875 and 2090 ddPSF (Fig 9) coincided with periods of reduced fish growth (low TGC), including the time of photoperiod manipulation used to induce parr-smolt transformation. On the other hand, enzyme activities were high at 2745 ddPSF, which coincided with a period of high growth. Of the protease enzymes, most attention has been given to a possible link between trypsin or ALP and fish growth, with relationships to enzyme activity and trypsin isozymes having been investigated in several studies [25,56–62]. Significant relationships have been reported in a number of growth trials that have used enzyme activity (as a surrogate for digestive capacity) and isozymes as indicators. Often, trials have involved the monitoring of growth over protracted periods with trypsin being monitored at the end of the trial, but in some studies there has been repeated monitoring of both trypsin and the growth trajectories of the fish to obtain an overview of temporal changes [57,60,62,63]. In our study there was a significant positive relationship between TGC in the period prior to sampling and the specific activity of trypsin recorded in the intestine (Fig 13A), but no significant relationships were found when the growth period subsequent to sampling and the period that spanned both preceding and subsequent growth were considered (Fig 13B and 13C). Although trypsin is secreted from the pancreas in response to the presence of nutrient-rich chyme in the upper intestine the enzyme activity we recorded must have reflected food intake over a period of several days, rather than trypsin secretion induced by ingestion of a single meal. That is, registrations of high specific activities of trypsin would not display links with good rates of growth, and vice versa, unless recorded enzyme activities reflected adaptations to patterns of daily feed intake by the fish over time. A relationship between feeding rates and digestive capacity has been demonstrated in Atlantic cod, with rapid and marked changes occurring in fish that are recovering from a prolonged period of food deprivation [59,64]. In other words, the specific trypsin activities we recorded at the different sampling times were likely a surrogate for digestive capacity (the potential of the digestive tract to hydrolyse protein). Intuitively, it would be expected that enzyme activity and digestive capacity would be influenced by, and reflect, feeding conditions and consumption in the period preceding sampling [59,64] and thereby act as an indirect indicator of previous growth (Fig 13A). This means that a direct cause-and-effect is being implied, with both digestive capacity and growth being influenced, or governed, by the amount of food consumed over a given time interval. The possibility exists that once established digestive capacity could act as a proxy to predict the subsequent growth of fish held under similar feeding conditions to those that were sampled. When this possibility was tested the lack of correlation between enzyme activity and growth (Fig 13B) served to cast doubt on the feasibility of using enzyme activity as a predictive indicator of subsequent growth and/or for growth over longer periods that span the time of sampling (Fig 13C). This conclusion seems to be supported by the findings of several previous studies. In some cases significant, but often weak, correlations between enzyme activities, digestive capacity and later growth have been found, but in others no such interrelationships were revealed [24,63,65]. As such, the results of our study not only confirm, but also expand, the findings of the relationship between intestinal tryptic activity and growth in salmonids, and under what conditions such a link may apply.

Weak relations might be a result of sampling procedures used for digestive enzyme analysis, because of the possibility of changes in the daily pattern of enzyme activity. To test this would require a comprehensive temporal analysis with samples being taken at different times after a meal for fish of each age, diet and ploidy. Gut morphology and diets can influence temporal patterns of gastric evacuation [23], digesta movement along the intestine, food hydration and gastrointestinal pH [66,67], and lead to variations in enzyme secretion patterns.

In fish with a stomach, enzymatic digestion starts here. The gastric glands secrete pepsinogen and hydrochloric acid, the latter reducing pH and inducing conversion of pepsinogen to pepsin. Activity of pepsin is often measured at pH 2.5 because the optimum activity of fish pepsins is generally within the pH range from 1.5 to 3.5 [23,68]. The diet can influence the pH through its buffering capacity arising from the proteins and minerals present [69]. Some authors suggest that the dietary buffering capacity is a major contributor to the increased pH of gastric fluids [70], reducing the efficiency of conversion of pepsinogen to pepsin. Exposure to low pH activates pepsinogen, the zymogen of pepsin, so assays carried out at pH 2.5 may reflect the activity of the total amount of zymogen present in a sample [23], rather than the peptic activity at the *in situ* gastric pH. For example, the pH optimum for Atlantic salmon pepsin has been reported to be 3.05, and an increase to pH 3.65 reduced peptic activity by ca 10% [71]. We examined peptic activity at pH 2.5 and at the pH recorded in the stomachs of the fish at the time of sampling (Table 5). The former probably reflects the potential total activity when pepsinogen is activated, and the latter reflects activity under recorded luminal conditions. Activity was lower when measured at the *in situ* pH than at pH 2.5, with the greatest reduction being observed when *in situ* pH was highest (Table 5). *In situ* gastric pH was lower, and peptic activity of the triploid salmon was generally higher than that of the diploids fed the same diets (Table 5). This implies that the initial stages of protein digestion may have been more effective in triploids than in diploids, and especially so in the triploids given the HFM diet, with lower buffering capacity than the STD diet. Exposure to low pH in the stomach, resulting from the secretion of hydrochloric acid, leads to separation of the lipid (fat) and protein fractions of the food. As such, the lower pH recorded in the stomachs of the triploid salmon may have enhanced the separation of these feed components, and the effect may have been greatest for the triploids given the HFM diet (Table 5).

Regarding intestinal enzymes, there were no clear and consistent patterns of differences in activity, neither between ploidies over time nor for fish fed a specific diet, although there was an indication that enzyme activities tended to be lower for fish given the STD diet (Fig 9). This trend was also observed for the diploid fish in the measurements made on the different regions of the digestive tract at 2745 ddPSF, particularly in pyloric caeca (Figs 11 and 12). At 2745 ddPSF enzyme activities in pyloric caeca of triploids were higher than those of diploids (Figs 11 and 12). As expected, activities of the intestinal proteases tended to be lower in the posterior intestine than in pyloric caeca (Figs 11 and 12), a finding that has been reported previously [71].

Alkaline phosphatase (ALP) may have several physiological roles relating to the digestion and absorption of nutrients and in maintaining the integrity of the intestinal wall [72]. It probably participates in the regulation of the absorption of lipids (fatty acids) and minerals, such as calcium and phosphorus. It also has a role in the control of bicarbonate secretion by the mucosa, thereby contributing to regulation of the pH of the mucosal surface [72]. In addition to its digestive functions, ALP, secreted by intestinal enterocytes, may play a role in intestinal homeostasis and protection, as well as in mediation of inflammation. For example, it has an effect on intestinal barrier function, and dephosphorylates the toxic, inflammatory lipopolysaccharides (LPS) of gram-negative bacteria. Exposure to microbial LPS leads to an upregulation of ALP, thereby preventing excessive inflammation and helping to maintain the integrity of the intestinal epithelial barrier [72–76]. ALP activity develops early in the ontogeny of fish, being present at hatching and developing quite rapidly thereafter [72]. As such, ALP activity would have been expected to have been well-established at the time of our first sampling at 875 ddPSF, and this was found to be the case (Fig 9). There may be regional differences in ALP activity, and in samples taken at 2745 ddPSF the specific activity of this enzyme was higher in the pyloric caeca than in the rest of the intestine (Fig 12). This was likely due to the substantial contribution of pyloric caeca to intestinal tract weight. Our findings conform to the pattern generally reported for fish, in which ALP activity shows a progressive decrease from the proximal to the posterior intestine [72]. ALP activity also appears to be influenced by feeding and the nutrient composition of the diet, with high ALP activity usually being observed in fish that are feeding well and are in good nutritional condition [59,72]. On the other hand, ALP activity decreases during periods of feed deprivation, but is rapidly restored once food again becomes available [72]. This seems to be confirmed by the results of our study, in which there were significant, albeit weak, relationships between specific activities of ALP recorded in the intestine and the growth metric TGC (Fig 14).

In our study, some differences in ALP activity were noted both between ploidies and in the fish fed the different diets (Fig 9) with ALP activities being higher in the fish given HFM than in those fed the STD diet and in triploids than in diploids. In previous studies ALP activity has been reported to be influenced by dietary protein concentration, type (e.g. animal or plant; whole proteins or hydrolysates) and amino acid compositions [72]. The use of protein hydrolysates as a feed ingredient has been reported to stimulate ALP activity in some studies carried out on the early life history stages of marine fish, but inclusion of protein hydrolysates at high concentrations may have an inhibitory effect on ALP [72]. Taken together, the results of our study on Atlantic salmon imply that there was a dietary influence either on the digestive functions of ALP and/or on the needs for maintaining the integrity of the intestinal wall.

The assesment of total alkaline protease activity can be used to assess digestive capacity but comparisons across studies are difficult because of differences in the preparation of enzyme extracts, and methods used to determine activity [33]. In our study, activities of the pancreatic proteases trypsin and chymotrypsin were higher in the pyloric caeca than the other gut sections (Fig 11). In salmon pancreatic tissue is concentrated in the region of the pyloric caeca. Trypsin and chymotrypsin are secreted into the anterior intestine in the pancreatic juice, and activities of these alkaline proteases tend to be high in the most anterior portions of the intestine. Dietary proteins may be digested and most of their AAs absorbed by the time the chyme reaches the posterior intestine [32,71,77]. Of the pancreatic proteases, trypsin is of particular importance because it has a role in activating a number of proenzymes, such as converting chymotrypsinogen to the active enzyme chymotrypsin [78]. As such, activities of the alkaline proteases, including chymotrypsin, might be expected to mirror those of trypsin but this was not always the case (Fig 9). For example, tryptic activity was much lower at 2090 ddPSF than at the preceding and subsequent sampling times, but chymotrypsin did not show an equivalent trough (Fig 9). A lack of concordance between the activities of trypsin and chymotrypsin has been reported in previous studies in which fish have been provided with feeds differing in nutrient composition, or held under conditions that have induced differences in feed intake and growth [34,35,57,58,62,78]. Although the reasons for the differences in the patterns of the enzymatic activities of trypsin and chymotrypsin remain unclear they have been linked to differences in growth trajectories and feed conversion observed in fish held under different conditions [24,57,61,63].

When data for the activities of the two pancreatic enzymes were used to calculate T:C activity ratios an interesting trend emerged (Fig 10). The numerical values were very similar for both ploidies over time, and at a given sampling point differences between fish given the STD and HFM diets were minimal. When the T:C data for the different sampling points, ploidies and feed types were combined with the growth (TGC) data two distinct clusters were seen. Low T:C values coincided with times of low rates of growth (low values for TGC), and vice versa (Fig 13). It is possible, therefore, that T:C could be used to provide an indication of the recent growth history of fish, as a predictor of future growth and as an indicator of growth over longer time periods for fish held under similar conditions.

This idea is not new, and there are several studies that provide some credence to the use of T:C as a growth indicator [56,57,61,63]. In our study, the data formed distinct clusters, rather than presenting a linear relationship between TGC and T:C, so T:C only provided a rather crude and imprecise indication of growth. On the other hand, significant relationships were recorded in plots of TGC against T:ALP (Fig 14). As such, T:ALP ratio may be a good alternative to T:C as a growth indicator.

The triploid salmon had lower ADCs than diploids for the essential and non-essential AAs that made up the proteins of both diets tested (Table 6). The digestibility of the AAs making up dietary proteins and peptides is likely to depend upon protease enzyme activity, absorptive capacity, or most probably a combination. Trypsin is a key enzyme for protein digestion because it acts both as a digestive enzyme and activates other proteases. This means that low tryptic activity may act as a limiting factor, leading to reduced protein digestion and amino acid bioavailability. This could provide a partial explanation for the reported links between trypsin and feed utilization and growth in salmonids and other fish species [56–60,65]. On the other hand, measurements of tryptic activity made on samples taken at 2745 ddPSF, at the same time that faeces were sampled for digestibility estimation, provided no indications of lower tryptic activity in the triploid fish than in the diploids. Similarly, there was no evidence that the activities of the other intestinal proteases were lower in triploids than diploids at this sampling point (Fig 9). As such, it is not possible to attribute the differences in amino acid digestibility between diploids and triploids to a lower intestinal protease activity in the latter. It is possible to hypothesize that morphological and physiological differences in the digestive tract of diploid and triploid salmon may play a role in determining the metabolic capacity of fish that differ in ploidy status. Triploidisation can trigger numerous effects on fish gut morphology such as reduced intestinal length and number of pyloric caeca in Atlantic salmon [39]. All these changes may affect the surface area and time available for nutrient absorption, and the nutrient transport across intestinal cell membranes. If so, such differences could explain the decrease in protein and AA digestibility in triploid fish observed in our study. Further work aimed at assessing the gastrointestinal evacuation time and digesta characteristics of diploid versus triploid salmon may provide valuable information about this aid and in the development of dietary formulations tailored to the needs of triploids.

Although there were some indications of higher proteolytic activities in the fish given the HFM diet, trends were not consistent and few significant differences were observed (Fig 9). Inclusion of HFM in fish feeds, including salmon feeds, has been reported to improve growth and feed utilization in some studies, but the data relating to digestibility and amino acid bioavailability are variable and inconclusive [29–32,34,35,37,79]. In our study with salmon the inclusion of HFM did not result consistent differences in proteolytic enzyme activities, nor was there improved digestibility and amino acid bioavailability of the HFM feed in either diploid or triploid fish (Table 6). No positive effects of HFM on dry matter, protein and energy digestibility in fish have been reported previously [80,81]. On the other hand, high dietary levels of hydrolysed protein or high percentages of free AAs may modify the rate of AA absorption compared to absorption rates of amino acids present in polypeptides or intact proteins [37]. This could result in a decrease of feed efficiency linked to the imbalance between patterns of absorption between protein-bound and free AAs [38,82].

Overall, we did not find any large differences in gut morphology and digestive enzyme activities between ploidies and diets under our experimental conditions. Future work should consider the daily patterns of enzyme activity and the way gut transit rates can be affected by diet composition and ploidy, and examine potential contributions to the development of improved aqua-feeds.

## Supporting information

S1 Fig Longitudinal sections of whole individuals of diploid (2n) and triploid (3n) Atlantic salmon at hatch. (A) Overview 2n individual. (B) Overview 3n individual. bc. bucco-pharyngeal cavity; pi. posterior intestine; es. esophagus; li. liver; mi. mid intestine; st. stomach; y. yolk sac. Scale. A-B. 1.75 mm.
(TIF)

S1 Table Three-way ANOVA for peptic activity (UA/g of fish) xdiet x ploidy x age (ddPSF). (DOCX)

S2 Table Three-way ANOVA for TAP activity (UA/g of fish) x diet x ploidy x age (ddPSF). (DOCX)

S3 Table Three-way ANOVA for trypsin activity (UA/g of fish) x diet x ploidy x age (ddPSF). (DOCX)

S4 Table Three-way ANOVA for chymotrypsin activity (UA/g of fish) xdietxploidyxage (ddPSF). (DOCX)

S5 Table Three-way ANOVA for LAP activity (UA/g of fish) x diet x ploidy x age (ddPSF). (DOCX)

S6 Table Three-way ANOVA for ALP activity (UA/g of fish) x diet x ploidy x age (ddPSF). (DOCX)
