# Supplementary figures and images for: Digestive tract morphology and enzyme activities of juvenile diploid and triploid Atlantic salmon (Salmo salar) fed fishmeal-based diets with or without fish protein hydrolysates
Source: PLoS One. 2021 Jan 11;16(1):e0245216. doi: 10.1371/journal.pone.0245216 (PMC7801030; doi:10.1371/journal.pone.0245216)

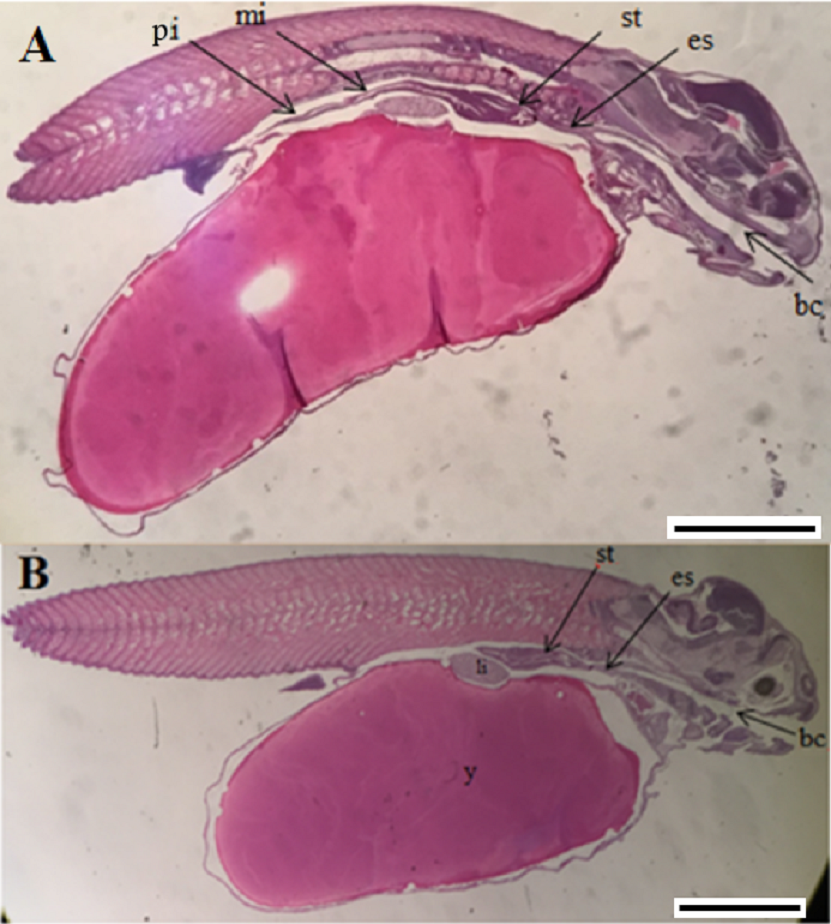

Supplement: S1 Fig — (A) Overview 2n individual. (B) Overview 3n individual. bc. bucco-pharyngeal cavity; pi. posterior intestine; es. esophagus; li. liver; mi. mid intestine; st. stomach; y. yolk sac. Scale. A-B. 1.75 mm. (TIF) [file pone.0245216.s001.tif]
