# Supplementary material for: Digestive tract morphology and enzyme activities of juvenile diploid and triploid Atlantic salmon (Salmo salar) fed fishmeal-based diets with or without fish protein hydrolysates
Source: PLoS One. 2021 Jan 11;16(1):e0245216. doi: 10.1371/journal.pone.0245216 (PMC7801030; doi:10.1371/journal.pone.0245216)
Supplement: S1 Table — (DOCX) [file pone.0245216.s002.docx]

**S1 Table. Three-way ANOVA for peptic activity (UA/g fish) x diet x ploidy x age (ddPSF)**

| **Source** | **Type III Sum of Squares** | **df** | **Mean Square** | **F** | **Sig.** |  |
| --- | --- | --- | --- | --- | --- | --- |
| *age* | 613,744 | 3 | 204581 | 83.19 | 0.0000 |  |
| *ploidy* | 34 | 1 | 34 | 0.01 | 0.9068 |  |
| *diet* | 19,087 | 1 | 19087 | 7.76 | 0.0059 |  |
| *agexploidy* | 69,037 | 3 | 23012 | 9.36 | 0.0000 |  |
| *agexdiet* | 33,539 | 3 | 11180 | 4.55 | 0.0043 |  |
| *dietxploidy* | 2,702 | 1 | 2702 | 1.10 | 0.2959 |  |
| *agexdietxploidy* | 11,119 | 3 | 3706 | 1.51 | 0.2143 |  |
| *Residual* | 442,643 | 180 | 2459 |  |  |  |
| *Corrected Total* | 1,191,560 | 166 |  |  |  |  |
| **Means by minimum square for peptic activity (UA/g fish) with 95% Confidence Interval (CI)** | | | | | | |
|  |  |  | **Error** | **Lower** | **Upper** |  |
| **Level** | **Number** | **Mean** | **Est.** | **Limit** | **Limit** |  |
| Global mean | 196 | 129.94 |  |  |  |  |
| *Age (ddPSF)* |  |  |  |  |  |  |
| 875 | 46 | 48.10 | 7.46 | 33.37 | 62.82 | a |
| 1455 | 46 | 145.61 | 7.32 | 131.17 | 160.05 | b |
| 2090 | 50 | 120.91 | 7.04 | 107.01 | 134.80 | b |
| 2745 | 54 | 205.16 | 6.75 | 191.84 | 218.49 | c |
| *Ploidy* |  |  |  |  |  |  |
| 2n | 102 | 130.36 | 4.92 | 120.64 | 140.08 |  |
| 3n | 94 | 129.52 | 5.18 | 119.30 | 139.75 |  |
| *Diet* |  |  |  |  |  |  |
| HFM | 97 | 119.99 | 5.12 | 109.89 | 130.08 | a |
| STD | 99 | 139.90 | 4.99 | 130.05 | 149.75 | b |
| *AgexDiet* |  |  |  |  |  |  |
| 875x2n | 26 | 49.20 | 9.73 | 30.01 | 68.39 | a |
| 1455x2n | 23 | 159.75 | 10.35 | 139.32 | 180.17 | b |
| 2090x2n | 26 | 136.91 | 9.75 | 117.67 | 156.16 | b |
| 2745x2n | 27 | 175.59 | 9.55 | 156.74 | 194.43 | b |
| 875x3n | 20 | 46.99 | 11.32 | 24.66 | 69.32 | a |
| 1455x3n | 23 | 131.48 | 10.35 | 111.05 | 151.90 | b |
| 2090x3n | 24 | 104.90 | 10.16 | 84.85 | 124.94 | ab |
| 2745x3n | 27 | 234.73 | 9.55 | 215.89 | 253.58 | c |
| *AgexPloidy* |  |  |  |  |  |  |
| 875xHFM | 21 | 36.71 | 11.14 | 14.72 | 58.69 | a |
| 1455xHFM | 23 | 154.46 | 10.35 | 134.04 | 174.89 | b |
| 2090xHFM | 25 | 111.48 | 9.99 | 91.76 | 131.19 | ab |
| 2745xHFM | 28 | 177.30 | 9.37 | 158.80 | 195.79 | b |
| 875xSTD | 25 | 59.48 | 9.93 | 39.90 | 79.07 | a |
| 1455xSTD | 23 | 136.76 | 10.35 | 116.34 | 157.19 | b |
| 2090xSTD | 25 | 130.33 | 9.93 | 110.75 | 149.92 | b |
| 2745xSTD | 26 | 233.03 | 9.73 | 213.84 | 252.22 | c |
| *DietxPloidy* |  |  |  |  |  |  |
| HFMx2n | 53 | 116.66 | 6.83 | 103.19 | 130.13 |  |
| HFMx3n | 44 | 123.31 | 7.62 | 108.27 | 138.36 |  |
| STDx2n | 49 | 144.07 | 7.10 | 130.06 | 158.08 |  |
| STDx3n | 50 | 135.74 | 7.02 | 121.89 | 149.59 |  |
