# Supplementary material for: Digestive tract morphology and enzyme activities of juvenile diploid and triploid Atlantic salmon (Salmo salar) fed fishmeal-based diets with or without fish protein hydrolysates
Source: PLoS One. 2021 Jan 11;16(1):e0245216. doi: 10.1371/journal.pone.0245216 (PMC7801030; doi:10.1371/journal.pone.0245216)
Supplement: S2 Table — (DOCX) [file pone.0245216.s003.docx]

**S2 Table. Three-way ANOVA for TAP activity (UA/g fish) x diet x ploidy x age (ddPSF)**

| **Source** | **Type III Sum of Squares** | **df** | **Mean Square** | **F** | **Sig.** |  |
| --- | --- | --- | --- | --- | --- | --- |
| *age* | 271,198 | 3 | 90399 | 39.57 | 0.0000 |  |
| *ploidy* | 8,199 | 1 | 8199 | 3.59 | 0.0597 |  |
| *diet* | 20,794 | 1 | 20794 | 9.10 | 0.0029 |  |
| *agexploidy* | 31,580 | 3 | 10527 | 4.61 | 0.0039 |  |
| *agexdiet* | 1,909 | 3 | 636 | 0.28 | 0.8408 |  |
| *dietxploidy* | 5,117 | 1 | 5117 | 2.24 | 0.1362 |  |
| *agexdietxploidy* | 12,425 | 3 | 4142 | 1.81 | 0.1463 |  |
| *Residual* | 427,204 | 187 | 2285 |  |  |  |
| *Corrected Total* | 778,763 | 166 |  |  |  |  |
| **Means by minimum square for TAP activity (UA/g fish) with 95% Confidence Interval (CI)** | | | | | | |
|  |  |  | **Error** | **Lower** | **Upper** |  |
| **Level** | **Number** | **Mean** | **Est.** | **Limit** | **Limit** |  |
| Global mean | 203 | 135.57 |  |  |  |  |
| *Age (ddPSF)* |  |  |  |  |  |  |
| 875 | 45 | 91.27 | 7.25 | 76.96 | 105.58 | a |
| 1455 | 48 | 145.78 | 6.91 | 132.15 | 159.42 | c |
| 2090 | 50 | 116.90 | 6.79 | 103.51 | 130.29 | b |
| 2745 | 60 | 188.32 | 6.17 | 176.15 | 200.49 | d |
| *Ploidy* |  |  |  |  |  |  |
| 2n | 106 | 142.00 | 4.66 | 132.81 | 151.20 | b |
| 3n | 97 | 129.14 | 4.94 | 119.39 | 138.88 | a |
| *Diet* |  |  |  |  |  |  |
| HFM | 100 | 145.81 | 4.87 | 136.20 | 155.43 |  |
| STD | 103 | 125.32 | 4.73 | 115.99 | 134.66 |  |
| *AgexDiet* |  |  |  |  |  |  |
| 875x2n | 25 | 111.52 | 9.57 | 92.65 | 130.40 | b |
| 1455x2n | 25 | 163.45 | 9.57 | 144.58 | 182.33 | c |
| 2090x2n | 26 | 111.76 | 9.40 | 93.21 | 130.31 | b |
| 2745x2n | 30 | 181.28 | 8.73 | 164.06 | 198.49 | d |
| 875x3n | 20 | 71.01 | 10.91 | 49.50 | 92.53 | a |
| 1455x3n | 23 | 128.11 | 9.98 | 108.43 | 147.79 | bc |
| 2090x3n | 24 | 122.05 | 9.79 | 102.73 | 141.36 | b |
| 2745x3n | 30 | 195.37 | 8.73 | 178.15 | 212.58 | d |
| *AgexPloidy* |  |  |  |  |  |  |
| 875xHFM | 21 | 103.99 | 10.74 | 82.80 | 125.17 |  |
| 1455xHFM | 24 | 158.10 | 9.79 | 138.78 | 177.41 |  |
| 2090xHFM | 25 | 121.83 | 9.63 | 102.83 | 140.83 |  |
| 2745xHFM | 30 | 199.34 | 8.73 | 182.13 | 216.56 |  |
| 875xSTD | 24 | 78.55 | 9.76 | 59.30 | 97.80 |  |
| 1455xSTD | 24 | 133.47 | 9.76 | 114.22 | 152.72 |  |
| 2090xSTD | 25 | 111.98 | 9.57 | 93.10 | 130.85 |  |
| 2745xSTD | 30 | 177.30 | 8.73 | 160.09 | 194.51 |  |
| *DietxPloidy* |  |  |  |  |  |  |
| HFMx2n | 55 | 157.33 | 6.46 | 144.59 | 170.07 |  |
| HFMx3n | 45 | 134.30 | 7.30 | 119.89 | 148.70 |  |
| STDx2n | 51 | 126.67 | 6.72 | 113.41 | 139.94 |  |
| STDx3n | 52 | 123.97 | 6.66 | 110.84 | 137.10 |  |
