# Supplementary material for: Digestive tract morphology and enzyme activities of juvenile diploid and triploid Atlantic salmon (Salmo salar) fed fishmeal-based diets with or without fish protein hydrolysates
Source: PLoS One. 2021 Jan 11;16(1):e0245216. doi: 10.1371/journal.pone.0245216 (PMC7801030; doi:10.1371/journal.pone.0245216)
Supplement: S3 Table — (DOCX) [file pone.0245216.s004.docx]

**S3 Table. Three-way ANOVA for trypsin activity (UA/g fish) xdietxploidyxage (ddPSF)**

| **Source** | **Type III Sum of Squares** | **df** | **Mean Square** | **F** | **Sig.** |  |
| --- | --- | --- | --- | --- | --- | --- |
| *age* | 1207.50 | 3 | 402.50 | 109.55 | 0.0000 |  |
| *ploidy* | 32.43 | 1 | 32.43 | 8.83 | 0.0034 |  |
| *diet* | 6.03 | 1 | 6.03 | 1.64 | 0.2016 |  |
| *agexploidy* | 127.08 | 3 | 42.36 | 11.53 | 0.0000 |  |
| *agexdiet* | 1.34 | 3 | 0.45 | 0.12 | 0.9475 |  |
| *dietxploidy* | 32.68 | 1 | 32.68 | 8.89 | 0.0032 |  |
| *agexdietxploidy* | 9.13 | 3 | 3.04 | 0.83 | 0.4800 |  |
| *Residual* | 690.76 | 188 | 3.67 |  |  |  |
| *Corrected Total* | 2129.76 | 166 |  |  |  |  |
| **Means by minimum square for Trypsin activity (UA/g fish) with 95% Confidence Interval (CI)** | | | | | | |
|  |  |  | **Error** | **Lower** | **Upper** |  |
| **Level** | **Number** | **Mean** | **Est.** | **Limit** | **Limit** |  |
| Global mean | 204 | 5.84 |  |  |  |  |
| *Age (ddPSF)* |  |  |  |  |  |  |
| 875 | 48 | 2.13 | 0.28 | 1.58 | 2.69 | a |
| 1455 | 48 | 8.22 | 0.28 | 7.68 | 8.77 | c |
| 2090 | 50 | 5.02 | 0.27 | 4.48 | 5.55 | b |
| 2745 | 58 | 7.97 | 0.25 | 7.48 | 8.47 | c |
| *Ploidy* |  |  |  |  |  |  |
| 2n | 107 | 6.24 | 0.19 | 5.87 | 6.61 | b |
| 3n | 97 | 5.44 | 0.20 | 5.05 | 5.82 | a |
| *Diet* |  |  |  |  |  |  |
| HFM | 99 | 6.01 | 0.19 | 5.63 | 6.40 |  |
| STD | 105 | 5.66 | 0.19 | 5.29 | 6.04 |  |
| *AgexDiet* |  |  |  |  |  |  |
| 875x2n | 27 | 2.45 | 0.37 | 1.72 | 3.18 | a |
| 1455x2n | 25 | 9.86 | 0.38 | 9.11 | 10.62 | c |
| 2090x2n | 26 | 5.22 | 0.38 | 4.48 | 5.96 | b |
| 2745x2n | 29 | 7.43 | 0.36 | 6.73 | 8.13 | c |
| 875x3n | 21 | 1.82 | 0.42 | 0.99 | 2.66 | a |
| 1455x3n | 23 | 6.59 | 0.40 | 5.80 | 7.38 | bc |
| 2090x3n | 24 | 4.81 | 0.39 | 4.04 | 5.59 | b |
| 2745x3n | 29 | 8.52 | 0.36 | 7.82 | 9.22 | c |
| *AgexPloidy* |  |  |  |  |  |  |
| 875xHFM | 22 | 2.38 | 0.42 | 1.56 | 3.20 |  |
| 1455xHFM | 24 | 8.44 | 0.39 | 7.66 | 9.21 |  |
| 2090xHFM | 25 | 5.05 | 0.39 | 4.29 | 5.81 |  |
| 2745xHFM | 28 | 8.18 | 0.36 | 7.47 | 8.89 |  |
| 875xSTD | 26 | 1.89 | 0.38 | 1.15 | 2.63 |  |
| 1455xSTD | 24 | 8.01 | 0.39 | 7.24 | 8.78 |  |
| 2090xSTD | 25 | 4.98 | 0.38 | 4.23 | 5.74 |  |
| 2745xSTD | 30 | 7.77 | 0.35 | 7.08 | 8.46 |  |
| *DietxPloidy* |  |  |  |  |  |  |
| HFMx2n | 54 | 6.82 | 0.26 | 6.30 | 7.33 | b |
| HFMx3n | 45 | 5.21 | 0.29 | 4.63 | 5.78 | a |
| STDx2n | 53 | 5.66 | 0.26 | 5.14 | 6.18 | a |
| STDx3n | 52 | 5.67 | 0.27 | 5.14 | 6.19 | a |
