# Supplementary material for: Digestive tract morphology and enzyme activities of juvenile diploid and triploid Atlantic salmon (Salmo salar) fed fishmeal-based diets with or without fish protein hydrolysates
Source: PLoS One. 2021 Jan 11;16(1):e0245216. doi: 10.1371/journal.pone.0245216 (PMC7801030; doi:10.1371/journal.pone.0245216)
Supplement: S4 Table — (DOCX) [file pone.0245216.s005.docx]

**S4 Table. Three-way ANOVA for chymotrypsin activity (UA/g fish) xdietxploidyxage (ddPSF)**

| **Source** | **Type III Sum of Squares** | **df** | **Mean Square** | **F** | **Sig.** |  |
| --- | --- | --- | --- | --- | --- | --- |
| *age* | 8.544 | 3 | 2.848 | 50.33 | 0.0000 |  |
| *ploidy* | 0.076 | 1 | 0.076 | 1.34 | 0.2488 |  |
| *diet* | 0.004 | 1 | 0.004 | 0.07 | 0.7853 |  |
| *agexploidy* | 0.405 | 3 | 0.135 | 2.38 | 0.0708 |  |
| *agexdiet* | 0.233 | 3 | 0.078 | 1.37 | 0.2518 |  |
| *dietxploidy* | 0.031 | 1 | 0.031 | 0.55 | 0.4589 |  |
| *agexdietxploidy* | 0.169 | 3 | 0.056 | 1.00 | 0.3964 |  |
| *Residual* | 10.639 | 188 | 0.057 |  |  |  |
| *Corrected Total* | 20 | 166 |  |  |  |  |
| **Means by minimum square for Chy activity (UA/g fish) with 95% Confidence Interval (CI)** | | | | | | |
|  |  |  | **Error** | **Lower** | **Upper** |  |
| **Level** | **Number** | **Mean** | **Est.** | **Limit** | **Limit** |  |
| Global mean | 204 | 0.73 |  |  |  |  |
| *Age (ddPSF)* |  |  |  |  |  |  |
| 875 | 48 | 0.54 | 0.03 | 0.47 | 0.61 | a |
| 1455 | 48 | 1.03 | 0.03 | 0.97 | 1.10 | b |
| 2090 | 50 | 0.52 | 0.03 | 0.46 | 0.59 | a |
| 2745 | 58 | 0.80 | 0.03 | 0.74 | 0.87 | b |
| *Ploidy* |  |  |  |  |  |  |
| 2n | 107 | 0.75 | 0.02 | 0.70 | 0.79 |  |
| 3n | 97 | 0.71 | 0.02 | 0.66 | 0.75 |  |
| *Diet* |  |  |  |  |  |  |
| HFM | 99 | 0.73 | 0.02 | 0.68 | 0.78 |  |
| STD | 105 | 0.72 | 0.02 | 0.68 | 0.77 |  |
| *AgexDiet* |  |  |  |  |  |  |
| 875x2n | 27 | 0.59 | 0.05 | 0.50 | 0.68 |  |
| 1455x2n | 25 | 1.09 | 0.05 | 1.00 | 1.19 |  |
| 2090x2n | 26 | 0.54 | 0.05 | 0.45 | 0.63 |  |
| 2745x2n | 29 | 0.75 | 0.04 | 0.67 | 0.84 |  |
| 875x3n | 21 | 0.49 | 0.05 | 0.39 | 0.59 |  |
| 1455x3n | 23 | 0.98 | 0.05 | 0.88 | 1.07 |  |
| 2090x3n | 24 | 0.50 | 0.05 | 0.41 | 0.60 |  |
| 2745x3n | 29 | 0.86 | 0.04 | 0.77 | 0.94 |  |
| *AgexPloidy* |  |  |  |  |  |  |
| 875xHFM | 22 | 0.57 | 0.05 | 0.47 | 0.67 |  |
| 1455xHFM | 24 | 0.98 | 0.05 | 0.88 | 1.08 |  |
| 2090xHFM | 25 | 0.54 | 0.05 | 0.44 | 0.63 |  |
| 2745xHFM | 28 | 0.84 | 0.04 | 0.75 | 0.93 |  |
| 875xSTD | 26 | 0.52 | 0.05 | 0.42 | 0.61 |  |
| 1455xSTD | 24 | 1.09 | 0.05 | 0.99 | 1.18 |  |
| 2090xSTD | 25 | 0.51 | 0.05 | 0.41 | 0.60 |  |
| 2745xSTD | 30 | 0.77 | 0.04 | 0.69 | 0.86 |  |
| *DietxPloidy* |  |  |  |  |  |  |
| HFMx2n | 54 | 0.76 | 0.03 | 0.70 | 0.83 |  |
| HFMx3n | 45 | 0.70 | 0.04 | 0.63 | 0.77 |  |
| STDx2n | 53 | 0.73 | 0.03 | 0.66 | 0.79 |  |
| STDx3n | 52 | 0.71 | 0.03 | 0.65 | 0.78 |  |
