# Supplementary material for: Digestive tract morphology and enzyme activities of juvenile diploid and triploid Atlantic salmon (Salmo salar) fed fishmeal-based diets with or without fish protein hydrolysates
Source: PLoS One. 2021 Jan 11;16(1):e0245216. doi: 10.1371/journal.pone.0245216 (PMC7801030; doi:10.1371/journal.pone.0245216)
Supplement: S5 Table — (DOCX) [file pone.0245216.s006.docx]

**S5 Table. Three-way ANOVA for LAP activity (UA/g fish) xdietxploidyxage (ddPSF)**

| **Source** | **Type III Sum of Squares** | **df** | **Mean Square** | **F** | **Sig.** |  |
| --- | --- | --- | --- | --- | --- | --- |
| *age* | 5.02E-02 | 3 | 1.67E-02 | 127.39 | 0.0000 |  |
| *ploidy* | 3.36E-04 | 1 | 3.36E-04 | 2.56 | 0.1114 |  |
| *diet* | 2.37E-04 | 1 | 2.37E-04 | 1.80 | 0.1811 |  |
| *agexploidy* | 1.73E-04 | 3 | 5.75E-05 | 0.44 | 0.7262 |  |
| *agexdiet* | 2.78E-04 | 3 | 9.27E-05 | 0.71 | 0.5500 |  |
| *dietxploidy* | 3.15E-08 | 1 | 3.15E-08 | 0.00 | 0.9877 |  |
| *agexdietxploidy* | 1.37E-04 | 3 | 4.56E-05 | 0.35 | 0.7913 |  |
| *Residual* | 2.47E-02 | 188 | 1.31E-04 |  |  |  |
| *Corrected Total* | 7.65E-02 | 166 |  |  |  |  |
| **Means by minimum square for LAP activity (UA/g fish) with 95% Confidence Interval (CI)** | | | | | | |
|  |  |  | **Error** | **Lower** | **Upper** |  |
| **Level** | **Number** | **Mean** | **Est.** | **Limit** | **Limit** |  |
| Global mean | 204 | 0.03289 |  |  |  |  |
| *Age (ddPSF)* |  |  |  |  |  |  |
| 875 | 48 | 0.02351 | 0.00168 | 0.02019 | 0.02682 | a |
| 1455 | 48 | 0.04547 | 0.00166 | 0.04220 | 0.04874 | b |
| 2090 | 50 | 0.01227 | 0.00163 | 0.00906 | 0.01548 | a |
| 2745 | 58 | 0.05030 | 0.00151 | 0.04733 | 0.05327 | b |
| *Ploidy* |  |  |  |  |  |  |
| 2n | 107 | 0.03159 | 0.00111 | 0.02940 | 0.03378 |  |
| 3n | 97 | 0.03418 | 0.00118 | 0.03186 | 0.03650 |  |
| *Diet* |  |  |  |  |  |  |
| HFM | 99 | 0.03397 | 0.00117 | 0.03168 | 0.03627 |  |
| STD | 105 | 0.03180 | 0.00112 | 0.02958 | 0.03402 |  |
| *AgexDiet* |  |  |  |  |  |  |
| 875x2n | 27 | 0.02275 | 0.00221 | 0.01840 | 0.02711 |  |
| 1455x2n | 25 | 0.04457 | 0.00229 | 0.04004 | 0.04910 |  |
| 2090x2n | 26 | 0.01157 | 0.00226 | 0.00712 | 0.01602 |  |
| 2745x2n | 29 | 0.04748 | 0.00213 | 0.04328 | 0.05168 |  |
| 875x3n | 21 | 0.02426 | 0.00253 | 0.01927 | 0.02924 |  |
| 1455x3n | 23 | 0.04638 | 0.00239 | 0.04166 | 0.05110 |  |
| 2090x3n | 24 | 0.01297 | 0.00235 | 0.00834 | 0.01760 |  |
| 2745x3n | 29 | 0.05313 | 0.00213 | 0.04892 | 0.05733 |  |
| *AgexPloidy* |  |  |  |  |  |  |
| 875xHFM | 22 | 0.02604 | 0.00249 | 0.02114 | 0.03095 |  |
| 1455xHFM | 24 | 0.04477 | 0.00235 | 0.04014 | 0.04940 |  |
| 2090xHFM | 25 | 0.01307 | 0.00231 | 0.00851 | 0.01763 |  |
| 2745xHFM | 28 | 0.05201 | 0.00217 | 0.04774 | 0.05629 |  |
| 875xSTD | 26 | 0.02097 | 0.00226 | 0.01652 | 0.02542 |  |
| 1455xSTD | 24 | 0.04618 | 0.00234 | 0.04156 | 0.05079 |  |
| 2090xSTD | 25 | 0.01147 | 0.00229 | 0.00694 | 0.01599 |  |
| 2745xSTD | 30 | 0.04859 | 0.00209 | 0.04446 | 0.05272 |  |
| *DietxPloidy* |  |  |  |  |  |  |
| HFMx2n | 54 | 0.03267 | 0.00156 | 0.02959 | 0.03575 |  |
| HFMx3n | 45 | 0.03528 | 0.00173 | 0.03187 | 0.03869 |  |
| STDx2n | 53 | 0.03052 | 0.00158 | 0.02740 | 0.03364 |  |
| STDx3n | 52 | 0.03308 | 0.00160 | 0.02993 | 0.03623 |  |
