# Supplementary material for: Digestive tract morphology and enzyme activities of juvenile diploid and triploid Atlantic salmon (Salmo salar) fed fishmeal-based diets with or without fish protein hydrolysates
Source: PLoS One. 2021 Jan 11;16(1):e0245216. doi: 10.1371/journal.pone.0245216 (PMC7801030; doi:10.1371/journal.pone.0245216)
Supplement: S6 Table — (DOCX) [file pone.0245216.s007.docx]

**S6 Table. Three-way ANOVA for ALP activity (UA/g fish) x diet x ploidy x age (ddPSF)**

| **Source** | **Type III Sum of Squares** | **df** | **Mean Square** | **F** | **Sig.** |  |
| --- | --- | --- | --- | --- | --- | --- |
| *age* | 181.45 | 3.00 | 60.48 | 41.85 | 0.0000 |  |
| *ploidy* | 23.78 | 1.00 | 23.78 | 16.45 | 0.0001 |  |
| *diet* | 64.94 | 1.00 | 64.94 | 44.94 | 0.0000 |  |
| *agexploidy* | 7.16 | 3.00 | 2.39 | 1.65 | 0.1790 |  |
| *agexdiet* | 1.43 | 3.00 | 0.48 | 0.33 | 0.8040 |  |
| *dietxploidy* | 11.55 | 1.00 | 11.55 | 7.99 | 0.0052 |  |
| *agexdietxploidy* | 1.12 | 3.00 | 0.37 | 0.26 | 0.8558 |  |
| *Residual* | 271.71 | 188.00 | 1.45 |  |  |  |
| *Corrected Total* | 569.47 | 166.00 |  |  |  |  |
| **Means by minimum square for ALP activity (UA/g fish) with 95% Confidence Interval (CI)** | | | | | | |
|  |  |  | **Error** | **Lower** | **Upper** |  |
| **Level** | **Number** | **Mean** | **Est.** | **Limit** | **Limit** |  |
| Global mean | 204 | 4.19 |  |  |  |  |
| *Age (ddPSF)* |  |  |  |  |  |  |
| 875 | 48 | 3.25 | 0.18 | 2.91 | 3.60 | a |
| 1455 | 48 | 5.27 | 0.17 | 4.92 | 5.61 | b |
| 2090 | 50 | 3.23 | 0.17 | 2.90 | 3.57 | a |
| 2745 | 58 | 5.02 | 0.16 | 4.71 | 5.33 | b |
| *Ploidy* |  |  |  |  |  |  |
| 2n | 107 | 3.85 | 0.12 | 3.62 | 4.08 | a |
| 3n | 97 | 4.54 | 0.12 | 4.29 | 4.78 | b |
| *Diet* |  |  |  |  |  |  |
| HFM | 99 | 4.76 | 0.12 | 4.52 | 5.00 | b |
| STD | 105 | 3.62 | 0.12 | 3.39 | 3.86 | a |
| *AgexDiet* |  |  |  |  |  |  |
| 875x2n | 27 | 3.23 | 0.23 | 2.77 | 3.69 |  |
| 1455x2n | 25 | 4.78 | 0.24 | 4.31 | 5.26 |  |
| 2090x2n | 26 | 2.72 | 0.24 | 2.25 | 3.19 |  |
| 2745x2n | 29 | 4.67 | 0.22 | 4.22 | 5.11 |  |
| 875x3n | 21 | 3.28 | 0.27 | 2.75 | 3.80 |  |
| 1455x3n | 23 | 5.75 | 0.25 | 5.26 | 6.25 |  |
| 2090x3n | 24 | 3.75 | 0.25 | 3.26 | 4.23 |  |
| 2745x3n | 29 | 5.38 | 0.22 | 4.94 | 5.82 |  |
| *AgexPloidy* |  |  |  |  |  |  |
| 875xHFM | 22 | 3.75 | 0.26 | 3.24 | 4.27 |  |
| 1455xHFM | 24 | 5.81 | 0.25 | 5.33 | 6.30 |  |
| 2090xHFM | 25 | 3.76 | 0.24 | 3.28 | 4.24 |  |
| 2745xHFM | 28 | 5.73 | 0.23 | 5.28 | 6.18 |  |
| 875xSTD | 26 | 2.75 | 0.24 | 2.29 | 3.22 |  |
| 1455xSTD | 24 | 4.72 | 0.25 | 4.24 | 5.21 |  |
| 2090xSTD | 25 | 2.71 | 0.24 | 2.24 | 3.18 |  |
| 2745xSTD | 30 | 4.31 | 0.22 | 3.88 | 4.75 |  |
| *DietxPloidy* |  |  |  |  |  |  |
| HFMx2n | 54 | 4.66 | 0.16 | 4.34 | 4.98 | b |
| HFMx3n | 45 | 4.87 | 0.18 | 4.51 | 5.22 | b |
| STDx2n | 53 | 3.04 | 0.17 | 2.71 | 3.37 | a |
| STDx3n | 52 | 4.21 | 0.17 | 3.88 | 4.54 | b |
